# Recent Advances in Synthesis and Properties of Hybrid Halide Perovskites for Photovoltaics

**DOI:** 10.1007/s40820-018-0221-5

**Published:** 2018-09-24

**Authors:** C. C. Vidyasagar, Blanca M. Muñoz Flores, Víctor M. Jiménez Pérez

**Affiliations:** 1 Universidad Autónoma de Nuevo León, Facultad de Ciencias Químicas, Ciudad Universitaria, Av. Universidad s/n, C.P., 66451 Nuevo León, Mexico; 2 School of Basic Sciences and Research in Chemistry, Rani Channamma University, PB NH-4, Bhutaramanahatti, Belagavi, Karnataka 591156 India

**Keywords:** Perovskites, Solar cells, Organic–inorganic perovskites, Synthetic routes, Fluorescence

## Abstract

The progress made by the scientific community in emerging photovoltaic technologies over the past two decades has been outstanding. Numerous methods have been developed for the preparation of hybrid organic–inorganic perovskite solar cells. The power conversion efficiency has been up to 14% by a one-step vacuum deposition technique. A serious concern is the toxicity of the materials. In this review, several methods aimed at resolving these problems to some extent have been compiled, including eco-friendly synthesis. Further efficiency enhancements are expected following optimization, and a better fundamental understanding of the internal electron charge transfer, electron–hole diffusion to the corresponding layers, flexibility, and stability-dependent bandgaps is reported. This paper explores the green synthesis of organic–inorganic perovskites for industrialization. Concerning the above facts, a simple low-cost model called “dispersed photovoltaic cells” is presented.
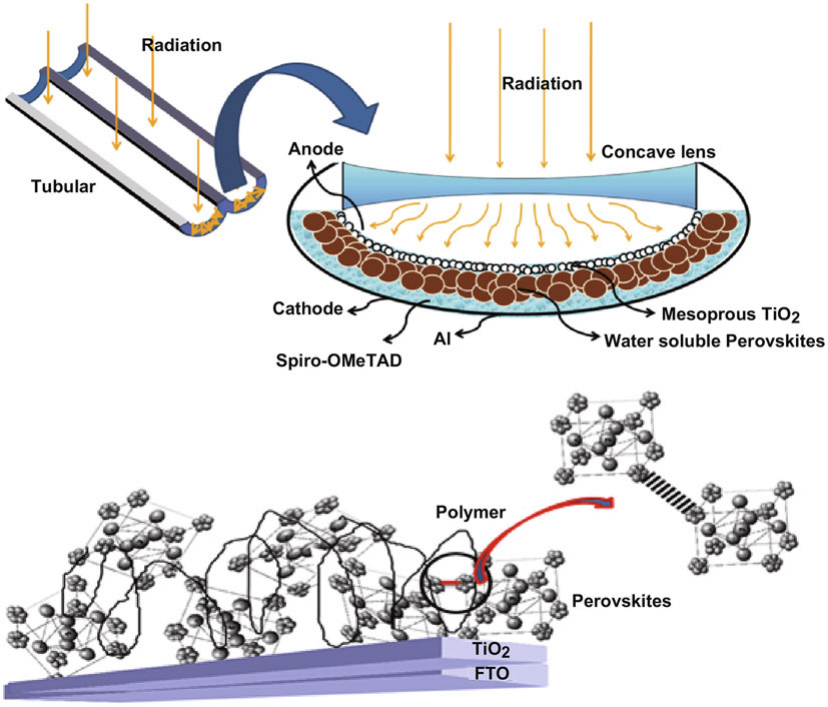

## Highlights

- Fourth-generation perovskite materials prevail in terms of efficiency (21%) and low-cost green synthetic routes.
- A serious concern is the toxicity of materials. Lead-free, highly efficient, relatively moisture stable, and environmentally friendly synthetic routes could be performed.
- Highly mobile charge carriers, low exciton binding energy, low rate of recombination, and charge transportation make perovskites a more challenging field.

## Introduction

**Fig. 1:**
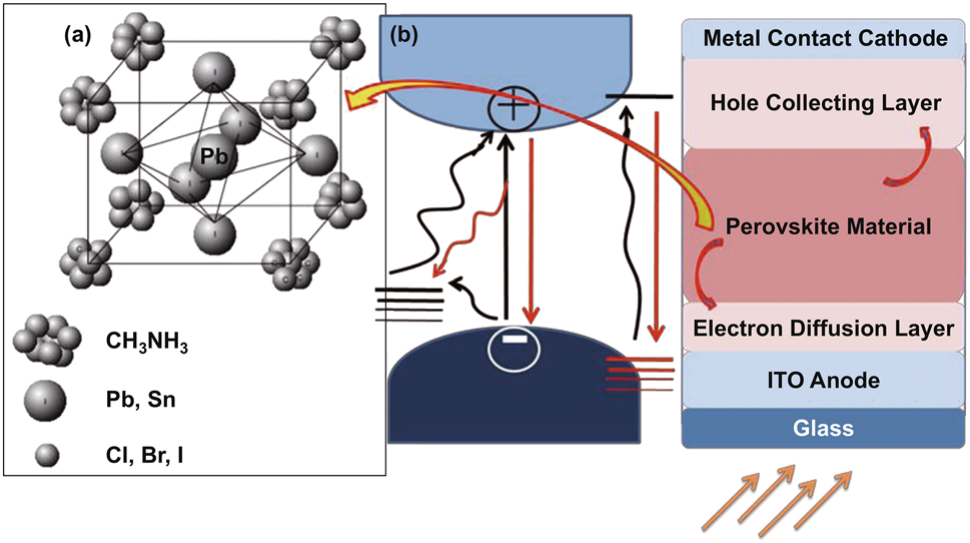
**a** Simulated crystal structure of CH_3_NH_3_BX_3_ obtained from the diffraction pattern and **b** electron–hole diffusion diagram in the material

Perovskite materials are a class of crystalline material with the formula ABX_3_, in which A and B are cations and X represents an anion. The ideal perovskite has a simple cubic crystal structure consisting of a corner-sharing BX_6_ octahedral network with a B–X–B bond angle of 180° and ions in the interstices (Fig. 1), and perovskite might be in orthorhombic phases by distortion of the BX_6_ octahedral at lower temperatures. The transition from orthorhombic to tetragonal to cubic perovskite structures was also found as a function of temperature. Organic–inorganic (OI) metal halide perovskite mesoscopic solar cells attract much attention owing to their ease in preparation, low cost, and high efficiency [1]. The immense interest in using these hybrid perovskites is because of their unique combination of properties that are critical for high photovoltaic (PV) performance: (1) a direct bandgap tunable by suitable choice of metals [2, 3], halogens [4], and organic cations [5]; (2) compared with organic polymers, they have a large dielectric coefficient, leading to minute exciton binding energy (20 meV) [6], extensive diffusion lengths, and lifetimes [7, 8]; (3) low-temperature solution process [9–11]; and (4) reliable absorption under the visible spectrum [12]. Hybrid OI semiconductors are opening up new insight into low-dimensional PV nanostructures. They bring a unique substituent of their organic and inorganic counterparts in devices and provide a significant opening for multifunctional materials for many electronic and optoelectronic applications. OI hybrids are thus a technologically important class of materials, offering the possibility of combining useful properties of organic and inorganic components within a single molecular composite. By modifying their molecular structures, it could be possible to change the optical and electrical properties of organic materials [13]. Despite significant study of these fundamental issues, it is clear that this system presents numerous unique challenges to researchers. Even so, a deeper understanding of these most basic processes will be a valuable asset to researchers aiming to push the performance of perovskite solar cells (PSCs) nearer the Shockley–Queisser limit and improve the stability of these devices beyond necessary commercial benchmarks [14]. Recently reported semiconducting perovskite materials, such as CH_3_NH_3_PbX_3_ (X = Cl, Br, I), could fulfill these requirements. A variety of nanostructured perovskite materials have high carrier mobilities and extended lifetimes with high PCE (> 9%) [15]. Figure 1 exhibits the possible two transitions at a higher wavelength (470 nm) and longer wavelength (770 nm) based on a dual valence band structure VB1 (470 nm) and VB2 (770 nm), so the charges could accumulate in a common conduction band minimum (CB1), regardless of the transition excited (via VB1 to CB1 or VB2 to CB1). The charge recombination dynamics are assumed to be homogeneous at all wavelengths. The first report of a solar cell incorporating a perovskite absorber was by Miyasaka et al. in 2009 and showed a 3.8% efficient perovskite-sensitized solar cell employing a liquid electrolyte [15]. This efficiency was further increased to 6.5% by Park et al. in 2011. However, due to the corrosive nature of the liquid electrolyte, the perovskite material was dissolved within a few minutes of device operation, which enhances a shift toward hole conductors. In the past 2 years, the efficiency of perovskite solar cells swept clearly from 10% to a certified 23.9% [15]. A perovskite/CIGS tandem configuration is an attractive and viable approach to achieve ultra-high efficiency and cost-effectiveness. Combining this cell in a mechanically stacked tandem configuration with a 16.5% CIGS cell results in a tandem efficiency of 23.9% [16].

Researchers at The Australian National University (ANU) have achieved a new record efficiency for low-cost semitransparent perovskite solar cells. The team led by the Duong from the ANU Research School of Engineering have achieved 26% efficiency by mechanically combining perovskite with silicon solar cells. The present research challenge is the substitution of Pb in the perovskite material with a nontoxic metal, but to date no scientific studies have been reported. Most recently, researchers have discovered a way to substitute Pb with a lesser amount of toxic material in perovskite-based solar cells. The most viable replacements for Pb in the perovskite material are tin and also members of the group 14 metals; thus, the major problem with the use of these metals is their chemical instability in the required oxidation state. Sn-based perovskites, in particular, have shown excellent features in solar cells, but can also be intentionally or unintentionally doped to become metallic. It has been demonstrated that when the Sn^2+^ ion is oxidized to Sn^4+^, the Sn^4+^ acts as a p-type dopant within the material in a process referred to as “self-doping.” CH_3_NH_3_SnI_3_ is a direct-gap semiconductor with an energy gap of 1.3 eV and a light absorption spectrum up to a wavelength of 940 nm, and it has been extensively used as a light harvester in solar cells [17]. Compared with conventional silicon-based solar cells, OI materials processed by vacuum and high-temperature-based thin-film fabrication techniques have become a center of attention [18]. However, thin-film fabrication techniques are relatively high cost in commercializing Pb-free OI perovskites. The Pb-free thin films could be fabricated by varying the doping level of CH_3_NH_3_SnI_3_. In turn, a carrier concentration of 1 × 10^19^ cm^−3^ has been achieved for the same, which reveals a strong p-type metallic character of a heavily doped semiconducting behavior. Ideally, all the possible synthetic methods should be tried in order to optimize individual materials to obtain better crystals with the proper microstructure. This is obviously time-consuming and very costly. Consequently, researchers usually choose to follow the general trends that have been observed to work in a particular area of interest [19–37]. The low-temperature solution-based film deposition techniques are needed to scale up the OI photovoltaic materials (Table 1).

**Table 1:** Compiled summary of the reported performance for the perovskite solar cells with synthesis and modification approach method

Films	Description	*J*_sc_ (mA cm^−2^)	*V*_OC_ (V)	PCE (%)	Refs.
CH_3_NH_3_PbI_3_/TiO_2_ films	Spin-coating method (solid-state device)	17	0.888	9	[19]
FTO/bl-TiO_2_/MAPbI_3_/Spiro-OMeTAD/Au	One-step spin coating, gas-assisted drying (300 nm)	22	1.05	17	[20]
FTO/bl-TiO_2_/mp-TiO_2_/MAPb (I_1−*x*_Br_*x*_)/PTAA/Au	One-step spin coating Solvent-engineering process (200 nm)	21.3	1.02	16.2	[21]
FTO/PEDOT:PSS/MAPbI_3−*x*_Cl_*x*_/PCBM/Al	One-step spin coating Hot-casting technique (450 nm)	22.4	0.92	17.7	[22]
ITO/PEDOT:PSS/MAPbI_3_/PCBM/C_60_/BCP/Al	Two-step spin coating in DMF solvent annealing (630 nm)	21.8	1.06	15.6	[23]
(CH_3_NH_3_PbI_3_·DMF and CH_3_NH_3_PbI_3_·H_2_O)	Gas–solid crystallization process	18.14	0.818	10.6	[24]
CH_3_NH_3_PbCl_3_/TiO_2_-compact-layer-coated FTO	Vapor-deposited perovskite, planar heterojunction thin film	21.5	1.07	15.4	[25]
CH_3_NH_3_PbCl_3_/TiO_2_/FTO	Solution-processed, planar heterojunction thin film	17.6	0.84	8.6	[26]
CH_3_NH_3_PbI_3_/TiO_2_ (mesoporous)	Microwave-synthesized	18.1	0.943	11	[27]
CH_3_NH_3_PbI_3_/JGC or P25 TiO_2_ (mesoporous)	Solvothermal-synthesized TiO_2_ 300 nm	19	1.03	11.8	[28]
CH_3_NH_3_PbI_3_ in DMF solvent/TiO_2_	Microwave-assisted synthesis, low power for 3 min (crystalline effect)	20.72	1.06	14.9	[29]
FTO/bl-TiO_2_/mp-Al_2_O_3_/MAPbI_2_Cl/Spiro-OMeTAD/Ag	Based on meso-superstructured organometal halide perovskites	17.8	0.98	10.9	[30]
CH_3_NH_3_)PbI_3_ [s]	Mechanosynthesis (ball milling)	15.2	0.778	8	[31]
(CH_3_NH_3_)PbI_3_/600 nm TiO_2_ film	Simple mixing solution process/spin-coated on HTM	7	0.645	2.5	[32]
MAPbSn_3_/Spiro-OMeTAD/Al_2_O_3_	400-nm-thick TiO_2_ spin-coated Spiro-OMeTAD	16.8	0.88	6.4	[33]
CH_3_NH_3_PbI_3_/Spiro-OMeTAD/mesoporous TiO_2_ scaffold	Solution mixing and spin-coated at different concentrations of (H-TFSI)	19.6	0.98	11.5	[32]
CH_3_NH_3_PbI_3_/mesoporous/Al_2_O_3_ scaffold	Solution mixing and spin-coated at different concentrations of (H-TFSI)	21.9	1.04	15	[32]
ITO/TiO_2_/CH_3_NH_3_PbI_3_	Solution deposition method (3:1) ratio CH_3_NH_3_I and PbI_2_ in DMF at low temp (150 °C)	19.9	1.06	13.8	[33]
ITO/Y-TiO_2_/CH_3_NH_3_PbI_3_ (different phase structures of TiO_2_)	Solution deposition method (3:1) ratio CH_3_NH_3_I and PbI_2_ in DMF at low temp (150 °C)	18.9	1.04	15	[33]
ITO/PEIE/TiO_2_/CH_3_NH_3_PbI_3_/Spiro-OMeTAD (PEIE-polyethyleneimine ethoxylated)	PEIE modification on ITO which changed the layer structure of the perovskite	22.8	1.15	19.3	[126]
Formamidinium tin iodide (FASnI_3_) in pyrazine mixture	Solvent-engineering and nonsolvent dripping process	23.44	0.28	4.3	[34]
Polyethylene naphthalate/ITO/Zn_2_SnO_4_	One-step solution process below 100 °C	21.6	1.06	15.3	[35]
TiO_2_/CH_3_NH_3_PbI_3_	Two-step deposition at higher temperature	21.27	1.03	15.7	[36]
Formamidinium lead iodide (FAPbI_3_) with methylammonium lead bromide (MAPbBr_3_)	One-step solution mixing and spin coating	22	1.08	17.3	[37]
FTO/bl-TiO_2_/mp-TiO_2_/FAPbI_3_/PTAA/Au	Fabricated through intramolecular exchange in DMSO	24.7	1.06	20.2	[33]
FTO/CuI/compressed SFP/PCBM/Al	Novel solvent-free perovskite deposition	22.13	0.85	7.7	[34]

## Disadvantages of Recent Solar Cells

**Fig. 2:**
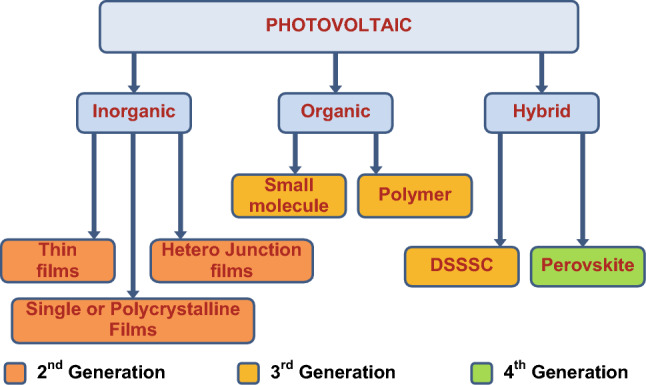
Classifications of photovoltaics corresponding to inorganic, organic, and hybrid perovskites

**Fig. 3:**
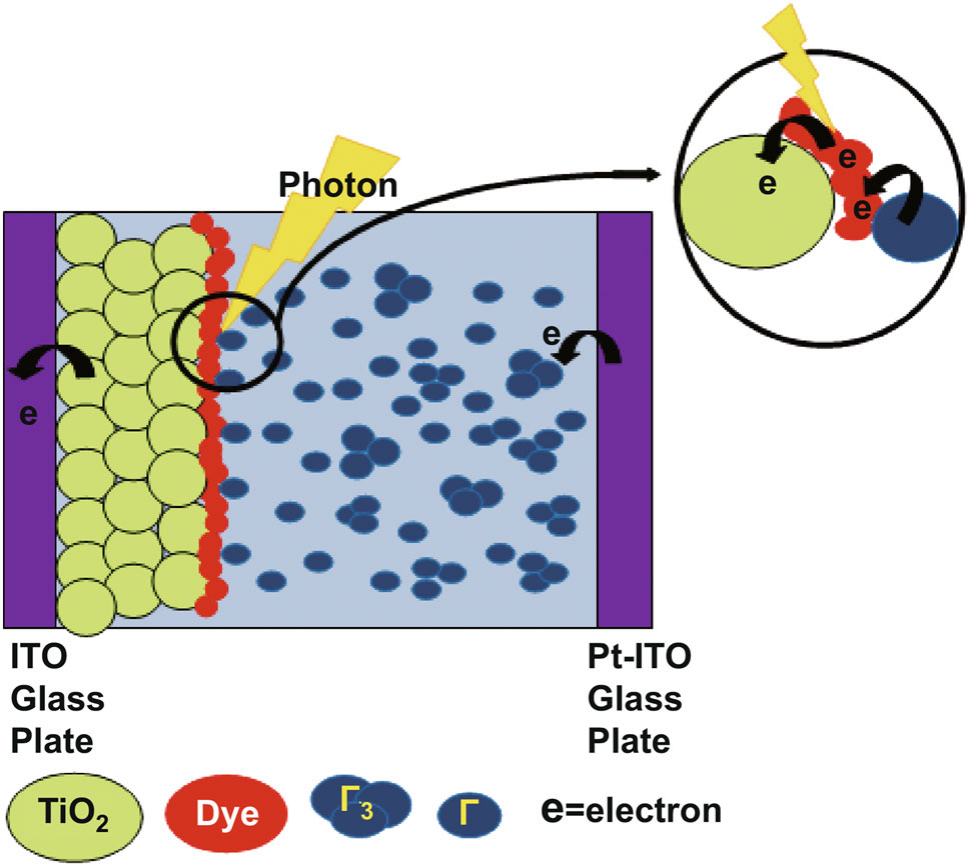
Working principle of dye-sensitized solar cells

Harvesting sunlight directly using photovoltaic technology is being recognized as the most essential and clean source of energy to meet future global energy demand. This generation of devices turned out to be advantageous in production cost with respect to silicon devices [38]. The thin films significantly achieved higher efficiency, because of different photon-absorbing layers. Because less energy is required and the owing to the relatively cost-effective fabrication of films, they can be fabricated economically in large areas. To date, single-crystalline silicon solar cells have shown promising PCE above 23% [38–40]. Now, the third-generation solar cells, such as dye-sensitized solar cells (DSSCs) and bulk heterojunction cells, are found to be good enough (Fig. 2) [38–41]. Furthermore, “third-generation” solar cells that are metal-free and have efficiencies of over 10% were developed [40–48]. Figure 3 shows the schematic electron transfer mechanism of dye-sensitized solar cells. Several researchers have reported the instability and the photodegradation of dyes, which slightly decreases the efficiency and negatively affects long-term stability [41, 49]. To overcome these problems, researchers reported the quantum dots, such as CdS [18], CdSe [50], PbS [51], InP [52], and InAs [53]. However, there is a considerable decrease in utilization of light and charge separation at the interface of the sensitizers [41, 54–57]. The GaAs, CdTe, and copper indium gallium selenide (CIGS) fabrication process lead to high cost and limit their wide application. Among all, perovskite solar cells are promising photovoltaic cells for inexpensive and large-scale solar energy conversion capability [58, 59] with cost-effective and high-throughput material capable of converting 18.2% of the solar energy to electricity, compared with an efficiency of 3.8% that was obtained only 4 years ago [59–61].

**Fig. 4:**
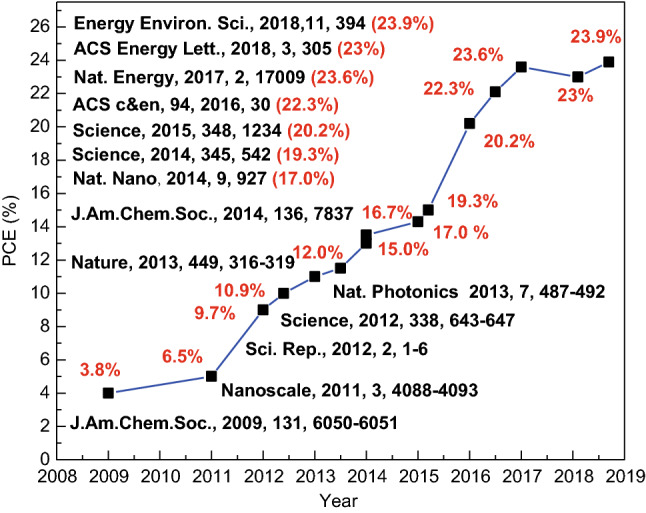
Stepwise progress in perovskite solar cells demonstrating the remarkably sharp rise in photovoltaic performance over the past 8 years

In the diverse field of solar cells, the selection of materials of merit is critical and, in some cases, is presented as a trade-off factor among durability, stability, cost, ease of fabrication, and efficiency. An ideal solar cell material requires inherent properties such as broader absorption covering the visible region, ultrafast carrier charge separation and transport high dielectric, and optimized diffusion lengths and low recombination rate. By extending its organic and inorganic features to the bottlenecked efficiencies of organic perovskites, the methylammonium metal halide perovskite becomes an important scientific breakthrough. An enormous number of elements in the periodic table are probably located at either A or B sites in the unit cell. This piece of evidence may provide a huge range of mixed compounds with structural similarities and a variety of properties. Among the most important properties are optical, electrical, and structural modifications that lead to the enhancement of solar cell performance. Kojima and Miyasaka first reported in 2009 on using CH_3_NH_3_PbBr_3_ and CH_3_NH_3_PbI_3_ as a sensitizer on porous TiO_2_ for the utilization of complete visible light in photoelectrochemical cells. The results revealed excellent optical and electrical properties. The cell performance was found to be 3.81% for CH_3_NH_3_PbI_3_ as compared with CH_3_NH_3_PbBr_3_. Afterward, Yang et al. reported that the best-performing flexible perovskite solar cells, based on the Zn_2_SnO_4_ (ZSO) and CH_3_NH_3_PbI_3_ layers, showed evidence of PCE of 14.85% under AM 1.5G 100 mW cm^−2^ illuminations. ZSO is well known as an n-type semiconductor as well as transparent-conducting oxide for optoelectronic applications with small electron effective mass and high electron hall mobility of 10–30 cm^2^ (V S)^−1^. Figure 4 shows an overview of the perovskite materials growing with time that has been compiled. These perovskite materials can not only serve as effective blocking layers, but they can also play an important role in the electron–hole transmittance to the corresponding electrodes. However, perovskite-based solar cells currently face several problems that hinder large-scale commercializing: (1) the toxicity of lead (Pb) atoms, (2) long-term stability, (3) effortlessness synthesis, and (4) recycling of the material. In this connection, future research should aim at finding highly efficient and environmentally friendly perovskite materials. Low-temperature solution-processed photovoltaics were found to have similar efficiency because of better crystallinity, electron–hole transfer, optical absorption length, and charge-carrier diffusion length.

**Fig. 5:**
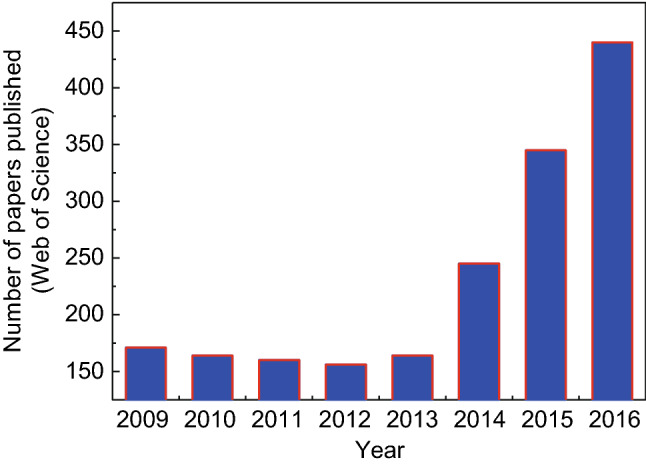
Yearwise progress in perovskite solar cell research published by various journals (data collected from Web of Science)

Although the efficiency of OI halide perovskite solar cells has shown rapid improvement within the past year, the toxicity has been tackled by the development of a mixed divalent metal perovskite. However, the issue of temperature stability seems to be linked to the organic cation utilized in perovskites, which may undergo a phase transition, even at slightly above the ambient temperature [62, 63]. The higher stability of the organic cations analog could arise due to a more rigid perovskite structure from the enhanced hydrogen bonding between cations and inorganic matrix. Nevertheless, there are also concerns about the potential environmental burden upon both occupational and nonoccupational exposures during fabrication and disposal of Pb-based perovskites. Many have proposed the replacement of Pb by nontoxic elements to render perovskite light absorbers. In addition, Pb is also listed as harmful chemical, raising concerns regarding its suitability as a more environmentally friendly alternatively to Pb in perovskite solar cells. In recent years, there has been increasing ecological and global public health concern associated with environmental contamination by these metals. Also, human exposure has risen dramatically as a result of an exponential increase in their use in several industries and technological applications. Apart from the metal toxicity issue, the use of the toxic solvent, for example DMSO and DMF, in the preparation of perovskites is another issue, as it will penetrate easily into the human body [58]. The present review compiles and directs researchers’ attention toward the necessary information to carry out a suitable procedure to synthesize perovskite compounds (Figs. 4, 5), and the collective information in this review gives guidance for current researchers and newcomers.

## Synthetic Routes of Perovskite

### Conventional Synthesis of Perovskites

Synthetic chemistry frequently consumes more time in trial-and-error experiments to standardize the methods. The more the steps, the more the attention should be paid to the reactions in order to minimize the impurities. There are a number of methodologies for fabrication of high-efficiency perovskite solar cells, such as the solution-processed two-step method [10, 64, 65], solution-processed single-precursor, and anti-solvent method [21, 37, 66], solution-processed adduct method [25, 67, 68], vacuum deposition method [69–71], ceramic solid-state reaction [72–74], glycine–nitrate route [75, 76], and sol-freeze-drying technique and combustion method [77–80]. Among them, the most promising method is the combustion method. Moreover, it is well known how difficult it is to tailor the properties in a one-step process at nanoscale for advanced technologies. In that case, the soft-chemistry routes surely are the most suitable strategies to pursue challenging objects [81]. However, most of the procedures suffer from requiring long reaction times, tedious work-up, and sufficient energy and consuming many liters of solvents. Indeed, combustion synthesis exploits exothermic reactions, which may reach up to the ignition temperature; thus, they do not need any additional energy to accomplish the production of the desired materials. Ignition of the exothermic reaction by a conventional heating technique reached a time range of minutes [82–84]. Rosa et al. [85] reported a comparison between conventional ignition and the microwave method. Preparation of perovskites requires significant amounts of energy in the process, because the ignition step and the energy-consuming steps reduce the unreacted reactants left in the reaction system. From the literature, significantly more uniform crystal sizes were obtained by exploiting microwave heating than by the combustion method.

### Hydrothermal Synthesis of Perovskites

Since the first application of hybrid OI perovskite materials on solar cells was reported in 2009, tremendous efforts have been made in the field. Some efforts focused on optimizing the physical and chemical properties. Recently, more studies have focused on structural properties, which they could correlate between crystalline and the chemical stoichiometry. So far, numerous methods have been investigated to prepare the CH_3_NH_3_PbX_3_ materials, such as spin coating [15], dip coating [64, 86], solid-state reaction [25], and precipitation reaction [87], but these precursors require the use of organic solvents, long reaction time, and careful adjustment of parameters. To date, an easy and rapid method has not yet been investigated to synthesize organometal halides. Solvents are used in mild conditions in the hydrothermal process, which permits rapid mixing of precursors for homogeneous products with controllable parameters. The hydrothermal technique has become one of the most adaptable chemical routes in terms of energy consumption, time-bound, and solvent-free methods. Peng reported a facile hydrothermal method to synthesize CH_3_NH_3_PbBr_3_ and CH_3_NH_3_PbI_3_. The final product was subjected to scanning electron microscopy (SEM) characterization, which results in rod-like structures without templates [88–93]. Wong et al. described low-temperature hydrothermal reactions to crystallize barium titanate and strontium titanate nanotubes. Barium titanate and strontium titanate (whichever is desired) and the synthesized TiO_2_ nanotubes (molar ratio 1:1) were mixed under Schlenk conditions to minimize CO_2_ contamination from the atmosphere, which may gradually lead to the formation of carbonate impurities [93]. The transmission electron microscopy (TEM) image reveals that the inner diameter of the nanotubes was 4 nm, and the outer diameter was 8 nm. The conventional hydrothermal preparation of nanohybrid materials with controllable reaction could achieve better morphology and crystal growth of particles. At severe treatment conditions above 350 °C at 50 MPa under different acidic or basic solutions, the lining material may undergo a rapid corrosion process. In addition, this method depends on the inorganic salt solubility in water under variable temperature and vapor pressure conditions. Use of organic cations in this method can be considered for careful pioneering experimentation to check its suitability for hydrothermal reactions. The effectiveness of the selected experimental approach can be evaluated by selecting the suitable chemical precursor concentration, pH of the media, temperature, and vapor pressure level, so this process is relatively complex and time-consuming due to numerous variables involved. Hence, a detailed state of the art regarding this technique should be focused on in the future.

### Solvothermal Synthesis of Perovskites

To investigate and overcome difficulties in crystal forms (crystal size and different phases), chemists achieved many milder reactions for homogeneous mixing of solid reagents. Among them, solvothermal synthesis is particularly well optimized for the preparation of inorganic solids and, most recently, metal–organic framework structures. Historically, hydrothermal synthesis was fine-tuned for the synthesis of homogeneous solid materials for industrial applications [94]. There are much fewer articles published for the one-step preparation of metal oxides using the solvothermal method. These functional materials are crucial for determining electronic, magnetic, photocatalysis, and high-temperature solid-oxide fuel cells because of various oxidation states [95, 96]. A few examples are chosen to show how the one-step solvothermal synthesis helps in nucleation and crystallization of materials, rather than using other conventional routes [97]. The perovskites containing transition metal have constructive properties arising from the control of specific oxidation states [98]. The functional materials synthesized by solvothermal and hydrothermal routes have been studied for crystallization [99]. Controlling crystal size and shape is the second challenging step by the use of solvothermal synthesis. Recently, a few studies showed the benefit of solution-mediated crystallization of oxides containing metals (lead, bismuth, or potassium) whose oxides are volatile, which can be avoided and maintain the stoichiometry of the precursors. All of these studies clearly showed how one-step solvothermal synthesis is becoming a powerful method for synthesizing materials at low temperature for different applications. In general, the majority of studies reported that crystallization of materials could be achieved by the use of solvents [100–104], because it is linked to the solubility of reactants. In most of the cases, solvent media are needed to bring the precursors for the nucleation process, which may lead to crystallization rather than using a high concentration of hydroxide salts. The major problems in developing the solvothermal routes could not be rationalized for all the materials. Thus, for solvothermal methods, more detailed understanding of the solution process is required. OI compounds prepared by conventional methods should have thermally stable metal salts and organic cations up to 400 °C at high vapor pressure. The effective heat transfer achieves the chemical reaction under solution conditions for conventional methods, and it is mentioned in this part that it is one of the main disadvantages. Most of the conventional hydrothermal and solvothermal reactions were conducted below the supercritical temperature of water, i.e., 374 °C. Thus, the microwave-induced hydrothermal and solvothermal methods have distinct advantages over conventional hydrothermal and solvothermal methods in the crystallization of different phases.

## Green Synthetic Routes

### Ultrasound-Assisted Synthesis of Perovskites

**Fig. 6:**
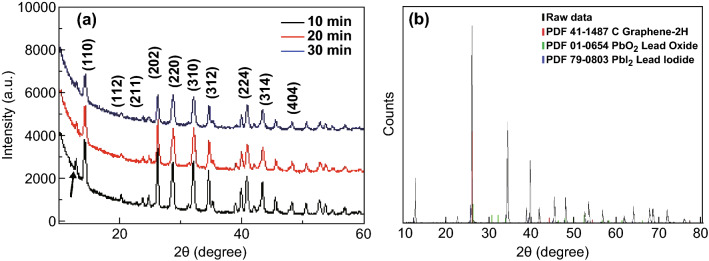
XRD patterns of CH_3_NH_3_PbI_3_ perovskite ultrafine nanoparticles: **a** after sonication in isopropanol for 10, 20, and 30 min (the peak assigned to unreacted PbI_3_ is marked) and **b** after 20-min sonication in water. Reproduced with permission Ref.  [109]. Copyright © 2016 Elsevier B.V.

**Fig. 7:**
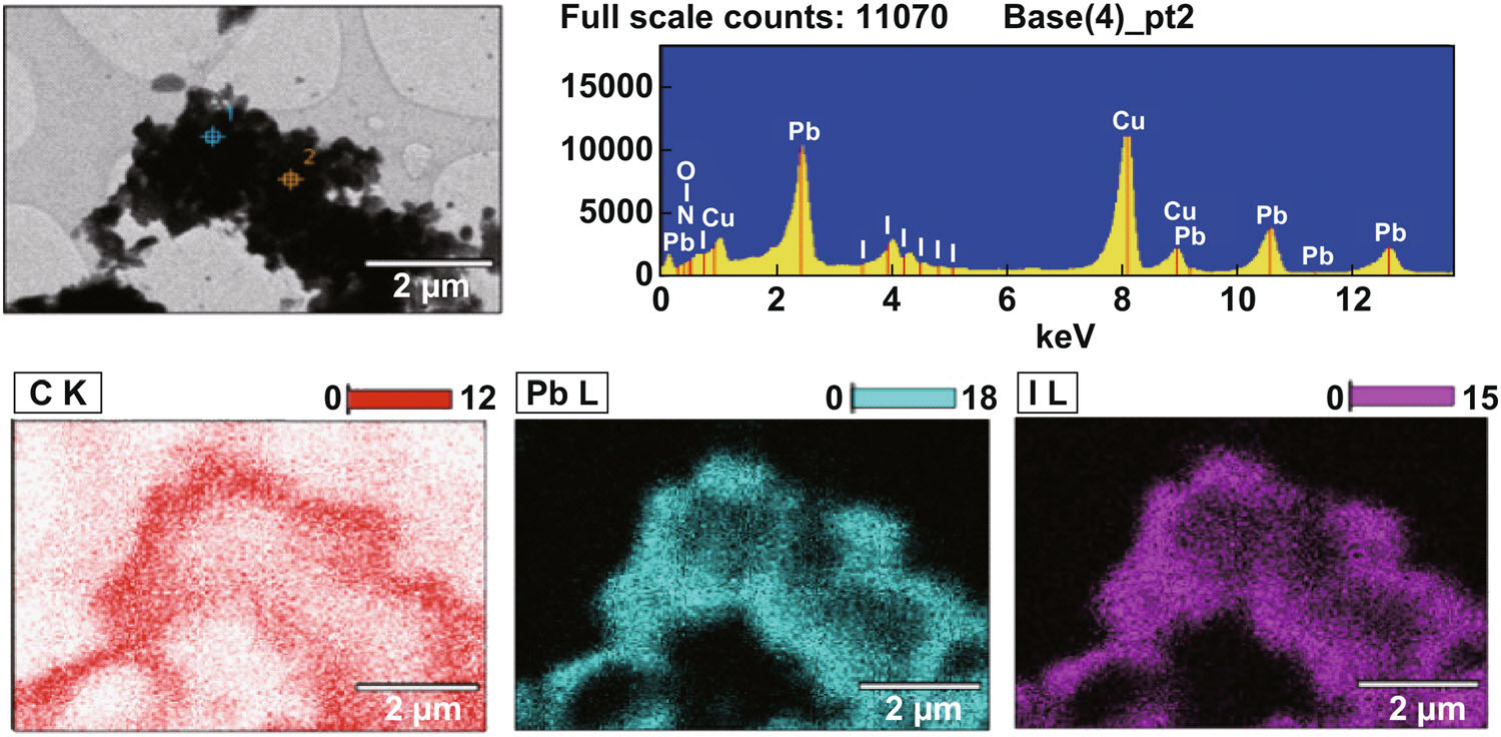
Plots of three consecutive measurements of DLS with Zeta Potential which is performed in isopropanol suspensions of the CH_3_NH_3_PbI perovskite at 20 min sonication [109]

**Fig. 8:**
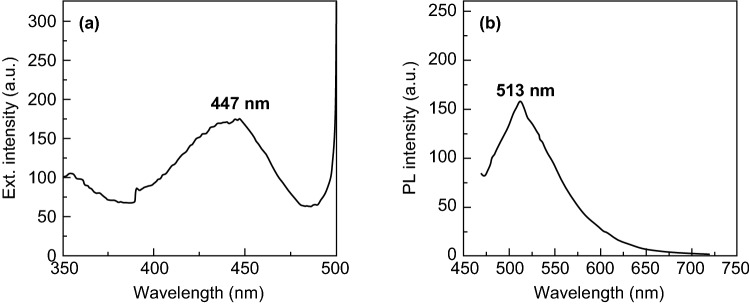
**a** Excitation spectrum and **b** emission spectrum of CH_3_NH_3_PbI_3_ nanoparticles. Reproduced with permission Ref.  [109]. Copyright © 2016 Elsevier B.V.

The fabrication of a stable quantum dot of perovskites was previously reported, but the commercial applications of quantum dot perovskites are still under investigation [21, 105–108]. Recently, an ultrasound-assisted synthesis of the perovskite in the range of 10–40 nm by the irradiation of CH_3_NH_3_I and PbI_2_, which were dissolved in isopropanol without any catalyst, has been reported [109]. To examine the effect of sonication, the solutions of two components were mixed by magnetic stirring at 40 °C using either regular laboratory stirring at 1000 rpm or high-speed stirring of approximately 6000 rpm (Ultraturrax device). The change in color from yellow to dark brown reveals the formation of CH_3_NH_3_PbI_3_, and the samples were characterized by X-ray diffraction (XRD), where the peaks of unreacted PbI_2_ were diminishing with the sonication time. The optimum sonication time was 20–30 min. No other peaks of precursors and PbO_2_ were detected in XRD (Fig. 6a), when water was used as the liquid medium for sonication instead of isopropanol (Fig. 6b). Figure 7 shows that the elemental analysis of particles was close to 20 nm. The excitation of the product was obtained at 447 nm (Fig. 8a), while the emission was observed at 513 nm (Fig. 8b). The material exhibits excitation-dependent emission performance only at an excitation wavelength longer than 430 nm, even by changing the excitation wavelength from 440 to 470 nm. The photoluminescence (PL) property changes with the different functional groups. The PL spectra showed a broad peak, which is attributed to the presence of a nonuniform distribution of particle size. It is postulated that, in many cases, a number of reports on conventional methods for the preparation of complex solid-state materials show a lack of detailed mechanistic understanding in terms of crystal growth, nucleation process, and crystal structure. There is a huge scope for the preparation of novel materials and novel methods, which have not yet been investigated. The conventional methods certainly make it difficult to understand several stable oxidation states. These problems may lead to the investigation of novel methods in the future to tailor the properties beyond the atomic-scale crystal structures.

### Microwave-Assisted Synthesis of Perovskites

It is well known that better crystals could be achieved by temperature tuning [68]. There are many reports of synthetic protocols for hybrid perovskites ranging from two-step procedures to direct crystallization [11, 27, 41]. It is difficult to control the nucleation and the crystallization from a saturated solution by evaporation of the solvent. The crystallization also depends on various parameters, such as an oxygen-rich atmosphere, and humidity leads to a mixture of crystal sizes and surface chemistries [17, 110]. In particular, the chemical and physical properties can be tuned at the atomic level by using reactants and thermal annealing. The most common thermal-annealing process for perovskite films is thermal annealing at 100 °C for 10 min. Subsequently, several alternative thermal-annealing processes were also developed to improve perovskite films, including a high-temperature thermal-annealing process and multistep thermal-annealing process. It is found that the perovskite materials obtained are crystallized faster with less energy of the microwave irradiation process [111–113]. The microwave irradiation process needs a specific material to have appropriate dipolar polarization and ionic conductivity to absorb microwave energy. Besides the common precursors, such as salt solutions of PbI_2_ and MAI based on coordinating solvents (DMF and DMSO), several low-dimensional intermediate states form poor crystallization [114]. It is expected that higher nucleation rates can be reached by accelerating the temperature. Therefore, microwave radiations are suitable to accelerate the temperature at the atomic level to get better crystallization.

**Fig. 9:**
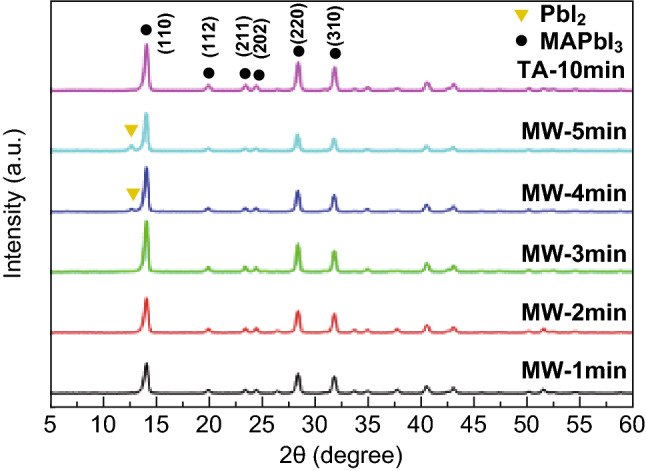
XRD pattern of perovskite films treated by 160-W microwave irradiation for 1, 2, 3, 4, and 5 min and annealed at 100 °C for 10 min. Reproduced with permission Ref.  [27]. Copyright ©2016 American Chemical Society

Using microwave radiation as a thermal initiator, the crystallization takes place within few minutes. Therefore, microwave synthesis appears to be a promising route toward the generation of hybrid perovskites [115–117]. Unless applying high power, dissolution does not take place, and the applied microwave power is crucial to the crystallization and efficiency (Table 2). As the microwave irradiation time increased, the crystallization of the material also increased, and the color also changed from white to yellow, and finally it became black, which was confirmed by XRD (Fig. 9) [9, 118–121]. However, the solvent plays an important role in preparation of perovskites under microwave irradiation. If there is no solvent residue, perovskite cannot form. The solvent might absorb the microwave irradiation energy and then transfer it to the perovskite powders, and thus, they are heated.

**Table 2:** Photovoltaic parameters of devices with different annealing processes

Samples prepared by microwave	*V*_oc_ (V)	*J*_sc_ (mA cm^−2^)	FF (%)	PCE (%)
MW-1 min	0.99	18.3	42	7.67
MW-2 min	1.06	19.07	63	12.64
MW-3 min	1.06	20.72	66	14.47
MW-4 min	1.05	20.24	61	12.99
MW-5 min	1.05	19.11	61	12.33
MW-10 min	1.05	20.69	64	14.02

**Fig. 10:**
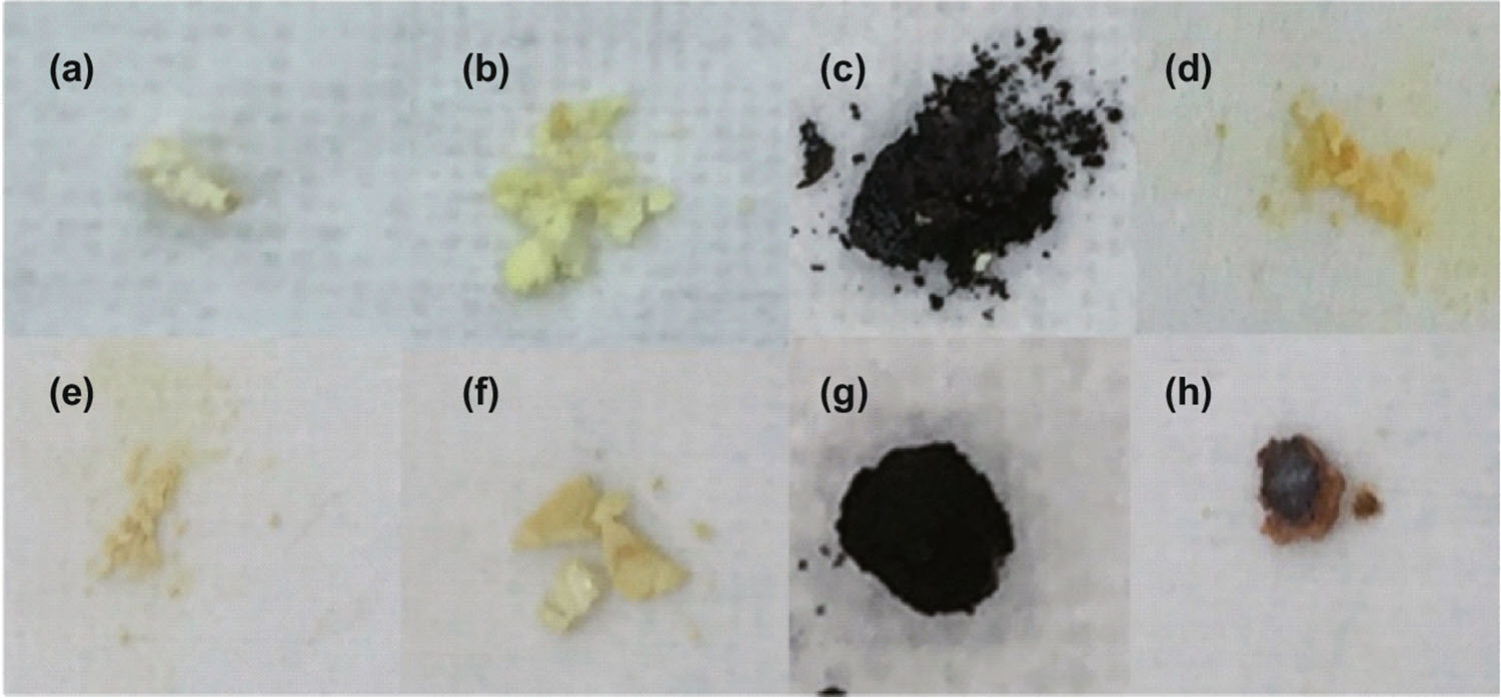
As-prepared perovskite material powder treated by microwave irradiation for **a** 0 min, **b** 1 min, **c** 2 min at 800 W and the PbI_2_–CH_3_NH_3_I adduct powders treated by microwave irradiation for **d** 0 min and **e** 2 min and the PbI_2_–CH_3_NH_3_I powders added with **f** diethyl ether, **g** DMF, and **h** DMSO treated by microwave irradiation for 2 min at 800 W. Reproduced with permission Ref.  [27]. Copyright ©2016 American Chemical Society

**Fig. 11:**
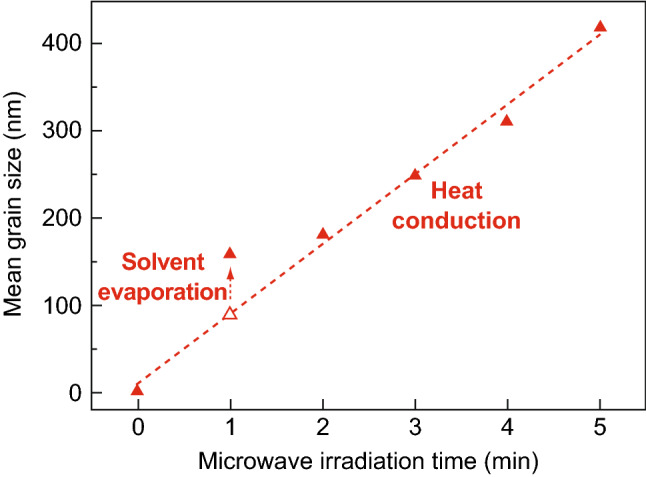
Time dependency of average grain size. Reproduced with permission Ref.  [27]. Copyright ©2016 American Chemical Society

To identify the role of solvents on perovskite materials under a microwave irradiation process, PbI_2_–CH_3_NH_3_I adducts were added to different solvents, such as DMF, DMSO, and diethyl ether. In Fig. 10f, it can be seen that adduct added to diethyl ether did not change to black under microwave irradiation. This implies that diethyl ether could not absorb the radiation and accelerate the perovskite precursor [27], but adduct added to DMF or DMSO turned black. The solvents absorb energy and convert it into heat during the vaporization of DMF or DMSO, which accelerate the precursors to react at the atomic level and crystallize. Therefore, the perovskite materials could rapidly crystallize with less energy loss and time consumption under appropriate solvents and microwave irradiation treatment. Figure 11 shows that the grain size of perovskite films showed a trend of linear increase when the time of microwave irradiation treatment varied with different time intervals. The grain size and crystallinity could be controlled at different time intervals. The results show the effect of crystallization, solvent, and morphology of perovskite films on the performance of solar cells. Thus, microwave irradiation is an effective mode of the process of synthesizing perovskite materials for solar cells.

### Mechanosynthesis of Perovskites

Grinding precursors are usually linked with old traditions in pharmacies, which are associated with the use of mortar and pestle. The past few decades have shown that effective solid chemical precursors can be used to synthesize desirable sizes and structures as a result of mechanical forces.

**Fig. 12:**
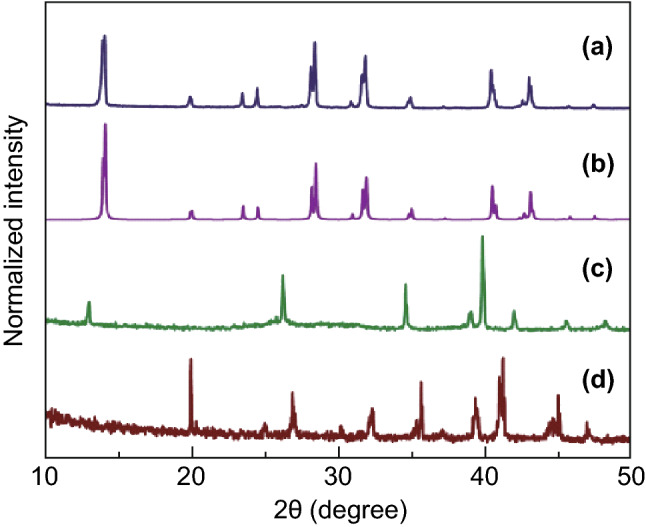
XRD patterns of as-prepared material: (a) simulated MAPbI_3_, (b) as-ground MAPbI_3_, (c) PbI_2_, (d) MAI. Reproduced with permission Ref.  [29]. Copyright © 2015 The Royal Society of Chemistry

**Fig. 13:**
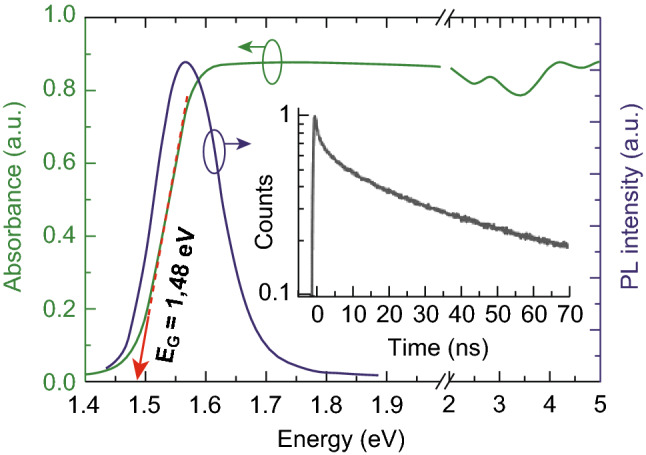
UV–Vis absorption (green peak) and PL (blue) spectra of as-ground MAPbI_3_ measured at ambient temperature: fluorescence decay excitation measured at the 3.05 eV (inset view). Reproduced with permission Ref.  [29]. Copyright © 2015 The Royal Society of Chemistry. (Color figure online)

**Fig. 14:**
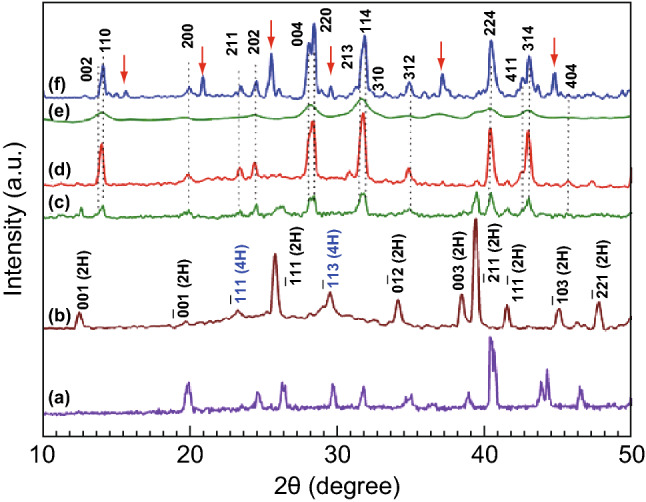
XRD patterns of (a) CH_3_NH_3_I, (b) PbI_2_, (c) ball milling for 10 min, (d) ball milling for 45 min, (e) CH_3_NH_3_PbI_3_/Al_2_O_3_, and (f) CH_3_NH_3_PbI_3_ by solution crystallization process. Reproduced with permission Ref. [135]. Copyright © 2016 Springer Science Business Media New York

Recently, Lewinski’s group from the Institute of Physical Chemistry of the Polish Academic of Science and Technology employed an effective method of preparing perovskites using a one-step mechanochemical process [122]. This process makes it possible to synthesize a variety of functional hybrid OI materials, which are potentially of great interest for various energy sectors. The mechanochemical method is the most environmentally friendly process of manufacturing the perovskites in the nanoscale region. Reactive grinding synthesis is applied to solid chemical reactions in which the phase transformation and size reduction process take place during milling by mechanical force [123, 124]. The mechanical force applied to the solid materials may undergo collision with the grinding media, which accelerates the development of more kinetic energy in the system [42–48, 125]. Different types of milling equipment, such as Spex shaker mill [89–91], planetary ball milling, attritor mills, and commercial mills, are used to produce mechanically alloyed powders by changing numerous parameters. Considering all the parameters, mechanosynthesis is a powerful tool to synthesis perovskites in an environmentally friendly, clean, and energy-efficient way [126–128]. It was stated by Kanatzidis et al. that simple grinding of MAI and PbI_2_ precursors in a mortar with a pestle results in significant quantities of unreacted precursors [2]. However, Gratzel and Lewinski reported that high-yield homogeneous perovskites could be obtained by neat grinding of MAI, and PbI_2_ results in polycrystalline methylammonium lead iodide perovskite particles and showed no detectable amount of the precursors in the products (Fig. 12) [29, 129–131]. Figure 13 shows the UV–Vis absorption and fluorescence spectra of CH_3_NH_3_PbI_3_ material. The measured peak reveals intense absorption over the entire UV–Vis region (green) with an absorption edge corresponding to 1.48 eV. Some small irregularity in the visible region refers to the presence of crystalline defects produced during the milling process, which could be the main parameter corresponding to the lower bandgap near the IR region. The photovoltaic factors of the best-performing devices are summarized in Table 3. They were prepared by a solvothermal and mechanochemical process [131–134]. In addition, Manukyan recently reported the mechanochemical synthesis of CH_3_NH_3_PbI_3_ perovskites. PbI_2_ and CH_3_NH_3_I powders were used as raw material in the ball-milling experiments [135]. CH_3_NH_3_I was synthesized by reacting CH_3_NH_2_ and HI, and the ball-to-mixture ratio was 10:1 from 200 to 600 rpm. The experiment was performed using planetary ball milling for 10–45 min and led to a change in color of the mixture. Figure 14 shows results of XRD analysis of the initial precursors PbI_2_ and the final product CH_3_NH_3_PbI_3_. Figure 14b shows that the peaks can be attributed to PbI_2_. After 10 min of reactive milling (Fig. 14c), the material showed peaks for the reactants and the products. Figure 14d reveals a single-phase and high-purity material, which was ground for 45 min. Figure 14e shows a similar strategy for the perovskite material, which was incorporated into mesoporous Al_2_O_3_ (Fig. 14f) [59, 130, 135–137].

**Table 3:** Summary of the efficiency of cells synthesized in two different methods

Perovskites	FF	*V*_oc_ (mV)	*J*_sc_ (mA cm^−2^)	Efficiency (%)
MAPbI_3_ (solvothermal)	68	778	15.2	8
MAPbI_3_ (mechanochemical)	72	879	14.2	8.9

Most recently, in 2014, Feng et al. demonstrated the crystal structures and the elastic and anisotropic properties of CH_3_NH_3_BX_3_ (B = Pb, Sn; X = Br, I) compounds in terms of strength of chemical bond B–X, in which they showed that organic cations are more crucial, and the interactions among organic and inorganic ions have an insignificant effect on elastic properties [138]. The experimental results imply that the organic and inorganic superlattices are effortlessly obtained by altering the ratio of organic and inorganic components and the method of solution preparation during crystallization. Furthermore, the complex structure defects and disordered CH_3_NH_3_^+^ ion lead to the phase boundary being unclear when different fabrication methods are used, and the experimental values agree well with the results calculated by empirical pairwise corrections in terms of a DFT + D^2^ scheme. The anisotropic of elastic properties are an important topic to be studied, having different effects on the anisotropic properties of the CH_3_NH_3_BX_3_ compounds. This anisotropic index is a better indicator than other indices and could provide unique and consistent results for the elastic anisotropy of perovskite compounds. However, different electronic properties, radii, and electronegativity of B^2+^ and X^−^ ions are essential for such observations. The results show that these perovskite compounds could be easily tunable by means of strain engineering in applications [139]. Most recently (2017), Cheetham et al. discussed the synthesis of formamidinium Pb halide perovskites (FAPbX_3_, X = Br or I) synthesized by inverse temperature crystallization to understand the effect of compositional engineering on their mechanical properties. They even examined the effect of the FA cation on the mechanical properties, in which the influence of the A-site cation is still a subject of discussion. Moreover, a few studies revealed that the organic molecule in a hybrid perovskite merely acts to balance and stabilize the charge and fill the space inside the 12-fold cavity and contributes slightly to the mechanical behavior. The results reveal that FAPbBr_3_ and MAPbBr_3_ both crystallize in the same cubic space group, making possible a direct evaluation between them. The Young’s moduli of FAPbBr_3_ are significantly lower than those of MAPbBr_3_ along all indentation directions. Replacing the MA cation with the larger FA cation increases the Pb–Br bond length by approximately 1% and weakens the inorganic framework. The hydrogen bonding is obvious, because more-pronounced pop-in events were observed in the bromide than the iodide perovskites. Their computational data show that FAPbBr_3_ exhibits a weaker hydrogen bond than MAPbBr_3_, and it was also found that the bond strength of the inorganic framework is a major factor determining the stiffness. The size of the organic cations becomes more important when the perovskites are at their limits of structural stability. Considering the consequence of the halide, the general consensus for the single perovskites is that the stiffness, which decreases as the electronegativity of the halogen decreases (Cl to Br to I), in turn reduces the Pb–X bond strengths in the cubic perovskites, which are the strongest as they lie along the direction of the Pb halide bonds [140, 141].

In the same year (2017), Markus et al. performed interesting studies on Goldschmidt tolerance and octahedral factors, which were found to be necessary geometrical concepts to evaluate the exact configuration of perovskite compounds, and the results were well in agreement with the reported hybrid compounds [142]. Taking into all these into consideration, the perovskite MAPbI_3_ as prepared by the mechanochemical approach has high efficiency with thermal stability. The newly synthesized perovskite material provided superior device performance compared with the standard solution process. This may open up new ways to synthesize inexpensive and high-performing perovskite materials for solar cells.

Here, we find that the green processes, such as microwave and mechanical synthesis, belong to green chemistry, and they are rapid, homogeneous, and relatively simple methods. The efficiency of the perovskites is also comparable to that of other methods. The mechanochemical reaction is a direct transfer of mechanical force to kinetic energy in terms of shearing, breaking, and stretching. Green synthetic methods have many advantages over typical synthetic methods involving chemical agents associated with environmental toxicity and consuming a greater number of times in trial and error when performing experiments. Green synthetic techniques have a great potential for preparing solid reactions without solvents or Schlenk line, lessening the reaction time and heating time, and also controlling product yields, which makes them cost-effective and environmentally friendly coordination compounds.

Direct reactive grinding synthesis is applied to solid chemical reactions in which the complex formation process takes place during milling by mechanical force. The reactive grinding process depends on the type of mill and the milling parameters. The mechanical force applied to the solid materials may undergo collision with the grinding media, which accelerates the development of more kinetic energy in the system. Mainly, the stress, applied force, and thermodynamic conditions could influence the complex formation, breakage of intramolecular bonds by external force in shearing Bridgman’s anvil or by friction at lubrication of rapidly moving cold contacting surfaces, and conformational changes of intermolecular ligations including hydrogen bonds, resulting in different yields of products. This may open up new ways to synthesize inexpensive and highly performing coordination compounds with relatively high yield, which are rapid, homogeneous, relatively simple methods. This type of synthesis is nowadays firmly established as a laboratory preparative procedure as well as a commercial production method. Table 4 summarizes the comparison of highest efficiencies achieved to date between conventional and green synthetic methods. Considering all the synthetic routes, green technology is a worthy and efficient room-temperature approach to scale up to produce typical hybrid OI perovskites by the mechanosynthesis method.

**Table 4:** Compiled data of the highest efficiency reported for the perovskite solar cells by conventional and green methods

Classifications	Synthesis	Perovskites	PCE (%)	Refs.
Conventional synthetic routes	Solution process method	Zn_2_SnO CH_3_NH_3_PbI_3_	14.85	[35]
	One-step spin-coating hot-casting	CH_3_NH_3_PbI_3−*x*_Cl_*x*_	17.7	[22]
	Gas–solid crystallization process	CH_3_NH_3_PbI_3_	10.6	[24]
	Vapor-deposited perovskite	CH_3_NH_3_PbCl_3_	15.4	[25]
	Solvothermal synthesis	CH_3_NH_3_PbI_3_	11.8	[28]
	Deposition method	CH_3_NH_3_PbI_3_	15	[33]
	Sequential deposition method	CH_3_NH_3_PbI_3−*x*_Cl_*x*_	13	[138]
Green synthesis	Mechanosynthesis	(CH_3_NH_3_)PbI_3_	9.1	[31]
	Microwave synthesis	CH_3_NH_3_PbI_3_	14.33	[29]

## Thin-film Perovskite Deposition and Stability Features

In the past 5 years, rapidly advancing techniques are becoming more attractive for the fabrication of hybrid Pb OI perovskites, because of their low-temperature processing, bandgap engineering, and high absorption coefficient. There are many reports regarding the film formation process of perovskites, including the deposition method and morphological optimization pathways. Some strategies and techniques, such as thermal annealing, solvent-free methods [34], conventional methods [28], solution process deposition [19–37], two-step spin coating [26], green synthesis [19–37], and doping or replacing with suitable metals, have been successfully employed to yield high-quality perovskites films with high efficiency. Despite the high efficiency, device stability is still poor for large-scale production. Thus, it is especially important to improve the stability for long durations of large-scale devices. Recently, methylammonium Pb halide perovskites demonstrated impressive progress due to their excellent absorption properties. In this study, one-dimensional PbI_2_/PVP composite fibers were prepared via an electrospinning process (Table 5) [143, 144]. The XRD analysis of PbI_2_/PVP composite fibers shows multiple crystalline phases, which indicates that PbI_2_ powder forms a composite fiber with PVP after dissolution and electrospinning without a high-temperature and high-voltage process. Polyvinylpyrrolidone plays an important role in the electrospinning process [145–148], and the morphology and diameter of the fibers depend on solution properties, such as viscosity (Fig. 13). These results are well in agreement with Chen et al. [31]. The dark dot in XRD shows that probably some part of PbI_2_ scattered in the PVP polymer could not react with MAI. This can be reduced by allowing the mixture to react with a high concentration of MAI [146–148].

**Table 5:** Different diameters of PbI_2_/PVP composite fibers obtained at different PVP concentrations, different applied voltages, and different spinning distances

Different PVP concentrations (wt%)	Mean diameter (nm)	Different applied voltages (kV)	Mean diameter (nm)	Different spinning distances (16 kV)	Mean diameter (nm)
3.08	180	12	221	2	284
3.58	212	14	196	4	273
4.07	238	16	211	6	257
4.55	195	18	199	8	254
5.03	270	20	185	10	211

Recently, it was found that device performance was strongly determined by the morphology and structure of the active layer, for which uncontrolled precipitation and large morphology variations lead to a nonuniform surface and less surface contact to corresponding electrodes, resulting in loss of performance. In two-step solution-processed spin coating, a layer of metal halide is deposited by spin coating followed by dipping the films into organic solution and drying at below 100 °C. In the dipping process, the rate of chemical reaction on the surface will be more and turns a different color immediately. However, it is observed that the reaction kinetics need to be controlled and developed for consistent device performance and reproducibility. Thus, in view of these problems, vapor deposition [149], co-evaporation [150–152], electrodeposition [153], and hybrid deposition [154, 155] techniques are much better in controlling the thickness and the reaction kinetics for the fabrication process of large-scale devices. Yang et al. developed a CH_3_NH_3_I vapor-based approach for the deposition of perovskite layers in a nitrogen environment. The approach is called a vapor-assisted solution process and achieved 13.84% efficiency at different (PbCl_2_/CH_3_NH_3_I) layers [156, 157]. The vapor deposition technique is widely used in semiconductor deposition in optoelectronics for large-scale production with controllable stoichiometry and morphology. Liu et al. [25, 31] reported that the synthesis of efficient perovskites using a dual-source vapor deposition technique with the precursors PbCl_2_ and CH_3_NH_3_I achieved 15.4%. Most recently, Lin et al. [158] reported that the use of vacuum-processed CH_3_NH_3_PbI_3_ perovskite with an ultrathin n-type and p-type layers cell had 16.5% efficiency.

Chang et al. [69] demonstrated a novel method of layer-by-layer sequential vacuum sublimation of perovskite fabrication. The process is comparatively simple, like the evaporation technique, in which the thin films are uniformly distributed with a high surface area. By incorporating the films with a hole transporter, poly(3,4-ethylenedioxythiophene) and poly(styrene sulfonate), and electron transporter, C_60_/bathophenanthroline, by thermal evaporation, an efficiency of 15.4% was obtained. Recently (2016), Liang et al., by controlling the morphology and crystallization of the films using one-step solution-processed chemical and physical vapor deposition (electrohydrodynamic assisted), recorded 16.6% (average 14.5%) [159]. Qi et al. used a hybrid chemical vapor deposition (CVD) method to fabricate films by thermal evaporation of PbCl_2_ followed by vapor-phase deposition of MAI, resulting in PCE of approximately 13.9% [160].

**Fig. 15:**
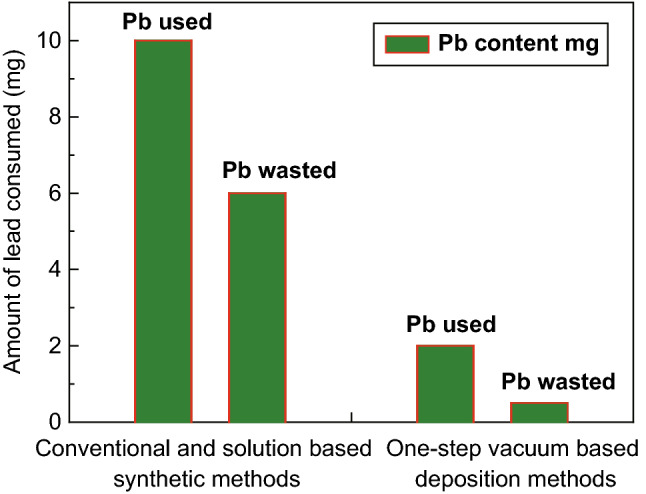
Consumption of lead can be significantly reduced by means of deposition and wasting throughout the experiment

In these physical deposition methods, the sublimation temperature of individual layers on the substrate significantly changes the morphology and the crystallinity of the layer, which also enhances the carrier transport between the layers. Sequential layer-by-layer vacuum deposition offers the possibility of minimizing the contamination and the possibility of getting a more uniform surface area (Table 6) [25, 161–172] and is a scalable method to fabricate efficient solar cells. In all fabrication routes and the development of highly efficient perovskites, Pb has been a major constituent to date. Because of concerns about environmental hazards and the disposal of Pb, relatively little was investigated to minimize the consumption of Pb during the deposition steps. Compared with the vacuum deposition route, to obtain the desired morphology and optimized coverage, spin coating, a solution process, and conventional routes need more Pb (100 μL) as a precursor (Fig. 15). However, more than 60–70% of solutions would be wasted throughout the experiments. In contrast, to reduce the Pb content in the fabrication process and industrialization, vacuum deposition methods could be better than the present synthetic ones in terms of stability, flexibility, efficiency, and long-term durability, which is relatively comparable to silicon-based solar cells. Clear and stable performance was observed in all the vacuum-based deposition methods (Table 6) for industrialization on a large scale.

**Table 6:** Summary of perovskite solar films fabricated by the different vapor-based techniques and corresponding PCE

Cell configuration and deposition techniques	Film thickness (nm)	*J*_sc_ (mA cm^−2^)	*V*_oc_ (V)	PCE (%)	Refs.
FTO/TiO_2_/CH_3_NH_3_PbI_3_Cl/spiro/Ag (co-evaporation)	330	21.5	1.07	15.4	[25]
ITO/polyTPD/CH_3_NH_3_Pb/PCBM/Au (co-evaporation)	285	16.1	1.05	12.04	[161]
ITO/polyTPD/CH_3_NH_3_I_3_/PCBM/TPYM/Au (co-evaporation)	285	18.2	1.09	14.8	[162]
ITO/PCDTBT/CH_3_NH_3_I_3_/PC_60_BM/LiF/Ag (co-evaporation)	250	21.9	1.05	16.5	[163]
ITO/MoO3/NPB/CH_3_NH_3_PbI_3_/C_60/_BCP/Al (co-evaporation)	320	18.1	1.12	13.7	[164]
FTO/C_60_/CH_3_NH_3_PbI_3_/spiro/Au (hybrid deposition)	320	18.9	1.1	15.7	[165]
FTO/C_70_/CH_3_NH_3_PbI_3_/spiro/Au (hybrid deposition)	320	18.6	1.03	14.9	[165]
FTO/TiO_2_/HC(NH_2_)_2_PbI_3−*x*_Cl/spiro/Au (hybrid CVD)	324	20.9	1.03	14.2	[166]
FTO/TiO_2_/CH_3_NH_3_PbI_3_/spiro/Ag (low-pressure CVD)	324	21.7	0.91	12.73	[167]
ITO/PEDOT:PASS/CH_3_NH_3_PbI_3_/Bphen/Ca/Ag (sequential deposition)	430	20.9	1.02	15.4	[69]
FTO/TiO_2_/CH_3_NH_3_PbI_3_/P_3_HT/Au (sequential deposition)	400	21.76	0.96	13.7	[168]
FTO/TiO_2_/CH_3_NH_3_PbI_3−*x*_Cl/spiro/Au (sequential deposition)	412	22.7	1	16.03	[169]
ITO/PEDOT:PASS/CH_3_NH_3_PbI_3_/polyTPD/PCBM/Ba/Ag (flash evaporation)	200	18	1.06	12.2	[170]
FTO/sputtered TiO_2_/CH_3_NH_3_PbI_3_/PbI_3−*x*_Cl_*x*_/HTM/Au (vacuum deposition)	500	22.76	0.96	12.29	[171]
FTO/c-TiO_2_/CH_3_NH_3_PbI_3_/pt (electrodeposition)	350	19.81	0.96	14.59	[172]

To date, many strategies have been exploited to improve the films’ performance in humid environments. In high humidity, the films decompose within a few hours, which results in worse film stability. A recent report showed that the peak absorption of films was reduced to 50% of the original value. Recently, Gratzel et al. improved the stability of the perovskite films with a phosphonic acid ammonium additive, which was cross-linked with the grain boundaries of the perovskite by hydrogen bonding [173, 174]. Zhao et al. fabricated them by using a polymer (polyethylene glycol) scaffold, which interconnects through the chemical bonds on films and works as a barrier to the high humidity.

Recently, Chen et al. demonstrated that the photostability and decomposition of perovskite films could be ended using ethyl cellulose (EC) [174]. EC-incorporated perovskites did not show degradation over 60% relative humidity for 5 days, and they were fabricated by a one-step spin-coating method [175]. The improved stability is attributed to the interaction between the perovskite and ethyl cellulose through hydrogen bonding. XRD confirmed that there is a slight change in the perovskite crystal structure by the addition of EC, but it was found that there is no structural change under 60% relative humidity in air and sunlight for 100 days. The cell represented as FTO/TiO_2_/CH_3_NH_3_PbI_3_/Spiro-OMeTAD/Au had 16.33% and 14.08% efficiency in the absence and presence of EC, respectively. The EC-incorporated perovskite showed a maximum UV–Vis absorption band compared with a bare one. In addition, the water treatment of perovskites without EC leads to a change in color from black to gray because of hydration, and it is reversible for several cycles, but the EC-incorporated samples did not change color. The results reveal that EC incorporation is an effective method to improve the stability of the films.

**Fig. 16:**
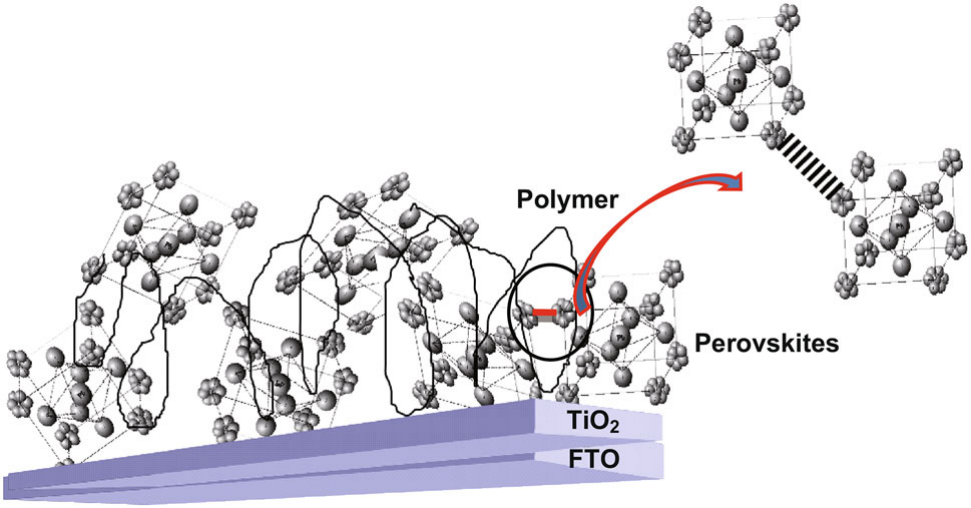
Schematic view of the CH_3_NH_3_PbI_3_ with ethyl cellulose

Jiang et al. reported the use of pseudohalide thiocyanate ions to replace two iodides from CH_3_NH_3_PbI_3_ to CH_3_NH_3_Pb(SCN)_2_I, which was stable for 4 h at 95% relative humidity [176]. The preliminary step involves the formation of hydrated intermediate, which decreases the binding between lead and halide. Further insight into the stability of the perovskites under moisture tolerance was confirmed by the bandgap test. The bandgap was decreased because of the degradation of the CH_3_NH_3_PbI_3_ structure compared with CH_3_NH_3_Pb(SCN)_2_I, and the presence of the SCN group was further confirmed by IR. Tai demonstrated the stability of the same perovskites by means of SCN incorporation into the CH_3_NH_3_PbI_3_ crystal lattice and achieved 15% efficiency under 70% relative humidity in ambient air for 500 h. They were fabricated by a two-step sequential deposition method [177]. This kind of testing is not only essential from the point of view of technology, but also it could provide further insights into the fundamental aspects and device designing of stable perovskites. More recently, Fakharuddin et al. demonstrated polymer-embedded perovskites (PVPs), which behave as barriers in the transfer of ions under relative humidity (Fig. 16) and avoid crystal defects by forming a bond between the perovskite crystals because of degradation [178]. The results reveal that degradation is caused by native surface defects of TiO_2_, which are activated in light and result in an imbalance of charges in the crystal lattice. This was further confirmed by the relative change in the crystal structure in the dark and light using Fourier transform infrared spectroscopy. By incorporating the polymer matrix (PVP), ion migration across the interfaces could be avoided.

So, by considering all the factors, it is possible to demonstrate how surfaces and interfaces can impact material properties and device performance. However, perovskite-based solar cells currently face several problems, such as long-term stability and the recycling of the material. For 3 years, vapor-based solution-processing techniques are becoming a subject for all researchers. The vapor-based methods require a certain level of vacuum, resulting in high cost. Therefore, more in-depth research is required for evaluating various fabrication methods for cost-effectiveness in a mass-production scenario.

## Flexible Appliances and Relative Moisture Stability Aspects

The whole world is looking for flexible appliances. An important aspect in electronic devices is their mechanical flexibility, which is essential for the development of bendable displays, sensors, biodegradable electronic devices, portable electronic chargers, and electronic boards and flexible cell phones. In the same way, solar cells too will be the subject of flexibility [179]. Flexibility will allow scaling up the production methods, such as roll-to-roll printing to lower the cost and power generation for a variety of electronics. In this regard, perovskites will show potential as flexible solar cells due to low-temperature processing (< 150 °C), because of solid-state structures, stability, and high efficiency. In recent years, flexible PSCs have made tremendous progress and achieved PCE of 15.3% (Table 7). Recently, Wang et al. [180] demonstrated flexible perovskite solar cells using TiO_2_ nanotubes arrays on Ti foils with transparent carbon nanotube as the electrode, yielding 8.31%. TiO_2_ nanotubes were prepared by electrochemical anodization at 20 V for 10 min at room temperature [180].

**Table 7:** Photovoltaic performance of some recent flexible perovskite solar cells

Cell configuration	*J*_sc_ (mA cm^−2^)	*V*_oc_ (V)	PCE (%)	Refs.
PEN-ITO/TiOx/MAPbI_3−*x*_Cl/Spiro-OMeTAD/Ag	21.4	0.95	12.2	[181]
PET-ITO/bI-TiO_2_/mp-TiO_2_/MAPbI_3−*x*_Cl_*x*_/Spiro-OMeTAD/Au	14.1	0.8	8.4	[185]
PET-ITO/ZnONps/MAPbI_3_/Spiro-OMeTAD/Ag	13.4	1	10.2	[182]
PET-ITO/Gr/ZnO QDs/MAPbI_3_/Spiro-OMeTAD/Ag	16.8	0.9	9.73	[186]
PET-ITO/Zn_2_SnO_4_/MAPbI_3_/PTAA/Au	21.6	1.05	15.3	[183]
PET-ITO/Ti/MAPbI_3_/Spiro-OMeTAD/Ag	15.24	0.83	8.39	[187]
Ti foil/bI-TiO_2_/Al_2_O_3_/MAPbI_3−*x*_Cl_*x*_/Spiro-OMeTAD/PET Ni	17	0.9	10.3	[188]
PET-ITO/PEDOT:PSS/MAPbI_3−*x*_Clx/PCBM/Al	16.5	0.86	9.2	[189]
PET-ITO/SOHEL2/MAPbI_3_/PCBM/Al	15.5	1.04	8	[190]
PET-ITO/PEDOT:PSS/MAPbI_3_/PCBM/bis-C_60_/Ag	14.62	0.86	9.43	[182]
PET/PEDOT:PSS/Ti/MAPbI_3_/PTCDI/Cr_**2**_O_3_/Cr/Au	18.5	0.97	13	[191]
PET-AZO/Ag/AZO/PEDOT:PSS/polyTPD/MAPbI_3_/PCBM/Au	14.3	1.04	7	[192]
PET/HC-PEDOT/SC-PEDOT/MAPbI_3_/PCBM/Al	15	0.8	7.6	[193]

Nowadays, the atomic layer deposition (ALD) method has been used for the fabrication of ultrathin uniform and conformal layers at low temperature in several PV technologies. In a recent report, Kim et al. [181] fabricated highly flexible (1-mm bending radius) mixed-halide perovskite (CH_3_NH_3_PbI_3−*x*_Cl_*x*_) on polyethylene naphthalate (PEN)-deposited ITO flexible substrate with a highest efficiency of 12.2% (Table 7). A TiO_2_ thin layer (20 nm) was deposited on PEN/ITO via a plasma-enhanced ALD technique. Its energy conversion factor did not change, even after 1000 cycles of bending testes under 10-mm bending radius. Compared with all the other deposition techniques, ALD seems better for the fabrication of flexible perovskite solar cells, which showed low degradation of efficiency during bending for 1000 cycles. This is due to the cracking of the ITO-PEN substrate at 4-mm bending radius, which decreased 50% of the cell performance. Kelly’s group initiated a thin film of ZnO NPs without sintering as an ETM both in the rigid PSCs and in the flexible PSCs, and it achieved approximately 15.7% and 10.2%, respectively. Later, they selected an alternative transparent conductive electrode to highly conductive poly(3,4-ethylenedioxythiophene): poly(styrene sulfonate) to ITO for the fabrication of PET-based flexible PSCs, and the device was ITO free and achieved PCE up to 7.6% [182].

**Fig. 17:**
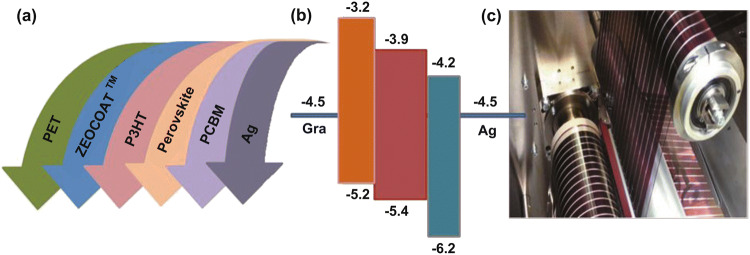
Roll-to-roll fabrication of perovskite photovoltaic sheets

Recently, highly dispersed Zn_2_SnO_4_ NPs prepared at low-temperature (< 100 °C) were introduced into the development of flexible PSCs with a peak PCE of 15.3%. The efficiency retained 95% of its initial value, even after 300 bending cycles, by the introduction of Zn_2_SnO_4_ film, which significantly improved the transmittance of the ITO-PEN substrate from 75 to 90% [183]. In addition, Shin et al. chose Ti instead of TiO_2_ as an efficient barrier layer to deposit directly on ITO-PET flexible substrate through RF magnetic sputtering and the resulting flexible PSCs without the metal oxide layer had PCE of 8.39%. There is a disadvantage of applying ITO or FTO on flexible substrates, because it degrades the cell performance due to cracks in the ITO and FTO layers upon bending [184–193]. Liu et al. [194] demonstrated that graphene can be successfully utilized as a flexible transparent electrode using a CVD method, due to its high transparency in a broad wavelength region. A single layer of graphene was deposited on copper foil followed by poly(methyl methacrylate) (PMMA) of 300-nm thickness. The cell configuration was polyethylene terephthalate/graphene/poly(3-hexylthiophene)/CH_3_NH_3_PbI_3_/PC_71_BM/Ag fabricated (Fig. 17), which showed a PCE of 11.48% with more bending radius (Fig. 17a, b).

**Fig. 18:**
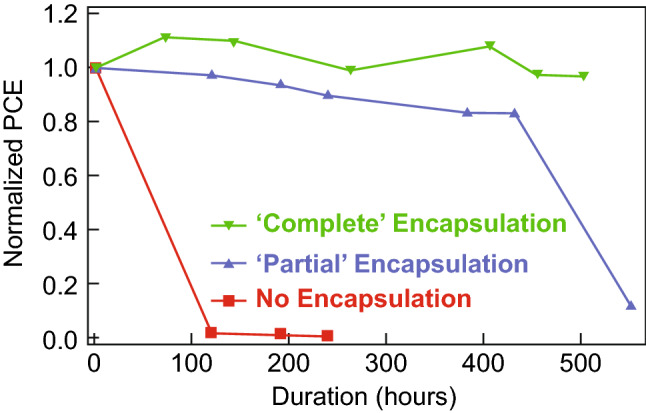
Normalized *I*–*V* parameter of nonencapsulation, partial encapsulation, and complete encapsulation perovskites. Reproduced with permission Ref.  [195]. Copyright © 2015 Elsevier Ltd.

**Fig. 19:**
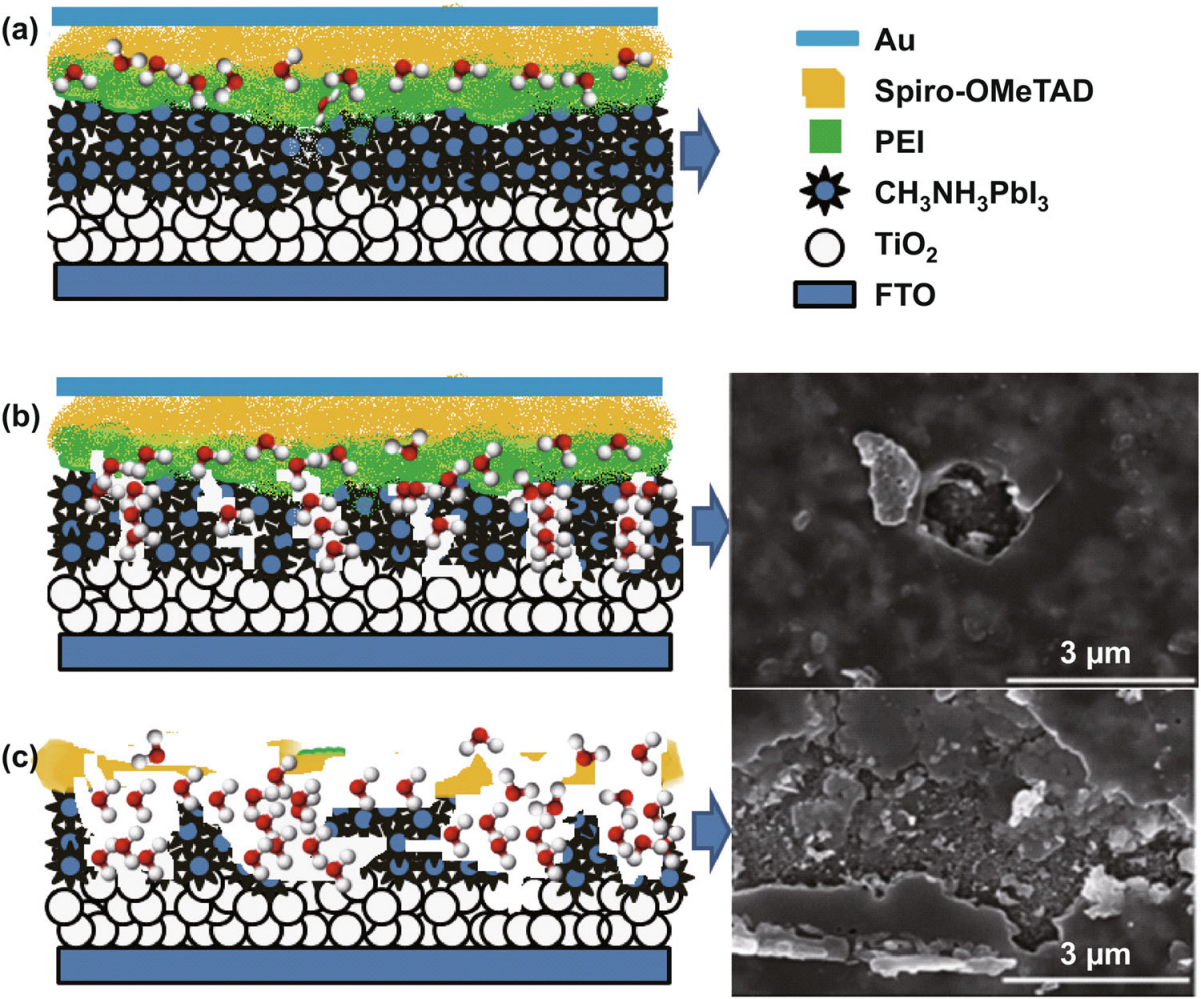
Top view of the uncoated Au perovskite cell: **a** cell with PEI layer, **b** degradation of perovskite in the form of nanopinholes with PEI layer, and **c** degradation of cell randomly all over without PEI layer and corresponding SEM images. Reproduced with permission Ref.  [196]. Copyright © 2015 The Royal Society of Chemistry

Hasitha et al. illustrated the effect of encapsulation to improve the operational lifetime of flexible solar cells fabricated on polymer substrate. The perovskite CH_3_NH_3_I and PbI_2_ (1:1) in DMF was coated using a spin coater in N_2_ atmosphere followed by deposition of OMeTAD in chlorobenzene. A transparent adhesive precoated tap of acrylic adhesive on poly-coated kraft was used as sealing material and as laminator to minimize the moisture content and oxygen to the perovskite material [195]. These results reveal that the encapsulated cell had good performance toward longer lifetime than without the encapsulation device (Fig. 18). Yun reported the improvement of the mechanical and moisture stability of the perovskites simultaneously by coating a layer of polyethyleneimine (PEI). Moreover, the PEI coating enhances the adhesion at the perovskite and hole transfer material (HTM) interfaces, which strongly adhere onto the perovskite and significantly prevent the degradation of CH_3_NH_3_PbI_3_ by moisture [196]. The PEI-coated cells were found to exhibit improvement in the mechanical and moisture stability on exposure to 85% relative humidity. The results revealed that strong binding of PEI at the interface of perovskite and HTM is superior to and more stable than that reported by Snaith and Wei [69, 71, 97, 154]. The organic polymer with amine groups will effectively bind to Pb metal, because carbon-based organic polymers show strong intermolecular attraction with the organic HTM layer. The samples coated with Spiro-OMeTAD of MAPbI_3_ with and without PEI were measured to be 1.44 and 3.23 ns, which obviously shows that the hole transfer from CH_3_NH_3_PbI_3_ to the HTM through PEI is more efficient than in the absence of PEI. One more point to be noted from the SEM images is that the decomposition performance for the cell with the PEI layer forms fragmentation in the order of nanopin holes (Fig. 19a, b) than the cell without the PEI layer (Fig. 19c). PEI is more hydrophilic than the CH_3_NH_3_PbI_3_, so the PEI layer strongly keeps the H_2_O molecule and will not allow it into the perovskite, in turn slowing down the degradation of the cell performance. Similar reports were also observed in Poorkazem’s work [182], where the incorporation of the hydrophilic layer on perovskite material delays the migration of H_2_O molecules into the cell. Incorporation of such an adhesive layer at the interface is crucial for the enhancement of mechanical and moisture stability of the perovskite cells. The film stability directly affected the photoconversion efficiency and retained the constant *J*_sc_ values while degrading.

According to recent publications, the vacuum deposition technique is the most effective in ensuring the flexibility and stability of Pb-free perovskites. The researchers tried to find a high stability and flexibility material using Pb-free elements, which are under screening by various groups, but have not yet been published anywhere else with acceptable results (Fe^2+^, Cu^2+^, Zn^2+^, Sn^4+^, Ti^4+^, Bi^3+^, Ge^2+^, and Sb^3+^) in terms of potential applications.

## Fluorescence Properties of Perovskites

Extensive attention has been drawn to the OI perovskite-material-based solar cells, which have a high photoconversion efficiency of 20%. The combination of exceptional optoelectronic properties, such as high mobile charge carriers, low exciton binding energy, and slow rate of recombination and charge transportation, all make perovskite a challenging field [89, 149]. An ideal solar cell material should have good optical absorption with efficient charge transport properties. The carrier diffusion and charge transporter in perovskite are major factors affecting the properties and performance of solar devices. The perovskites prepared by low-temperature solution processing and chemical bath deposition are amorphous or have a poor crystalline nature, suffering from poor charge-carrier transport. Recently, CH_3_NH_3_PbI_3_ material extensively used in solid-state solar cells exhibited an impressive 15% efficiency. Kim et al. [197] reported that the thin layer of perovskite material was sandwiched by a mesoporous TiO_2_ photoanode and a hole-transporting layer (2,2′,7,7-tetrakis(*N*,*N*-di-*p*-methoxyphenylamino)-9,9-spirobifluorene [8, 198]. However, Lee et al. showed that highly efficient perovskite materials could be achieved by replacing mesoporous TiO_2_ with an Al_2_O_3_ layer, involving good electron transport with 5.5% efficiency [89, 152]. Later, Etgar et al. [199] reported the same efficiency of 5.5% cell performance without using a hole-transporting layer. Ball et al. revealed that a 350-nm-thick layer sandwiched between TiO_2_ could generate a short-circuit current of 15 mA cm^−2^ [132]. Xing et al. reported that the CH_3_NH_3_PbI_3_ material was sandwiched by two different layers: one is electron extraction, such as {[6,6]-phenyl-C_6_-butyric acid methyl ester} [PCBM], and the other is a hole extraction layer, such as {poly(3,4-ethylenedioxythiophene)poly(styrenesulfonate)} [8, 200]. Comparing the bare CH_3_NH_3_PbI_3_ and CH_3_NH_3_PbI_3_/electron and hole extraction bilayer measurements, it is possible to identify the electron-transport characteristics in the OI halide. PL quenching suggests that the charge-carrier diffusion length inside the CH_3_NH_3_PbI_3_ layer is longer than the layer thickness (65 nm). Correspondingly, the PL lifetimes were also substantially shortened when CH_3_NH_3_PbI_3_ was interfaced with the PCBM. To improve the accuracy of these materials on the photoexcited charge carriers, transient absorption spectroscopy (TAS) was performed. Xing et al. [8] reported that the lifetime of thin films was 4.5 ns, and Stranks et al. [201] observed that the same was 9.6 ns. Yamada et al. [202] reported that the lifetime at low excitation absorbance was 140 ns, which may slightly increase in the case of single crystals around 100 μs.

**Fig. 20:**
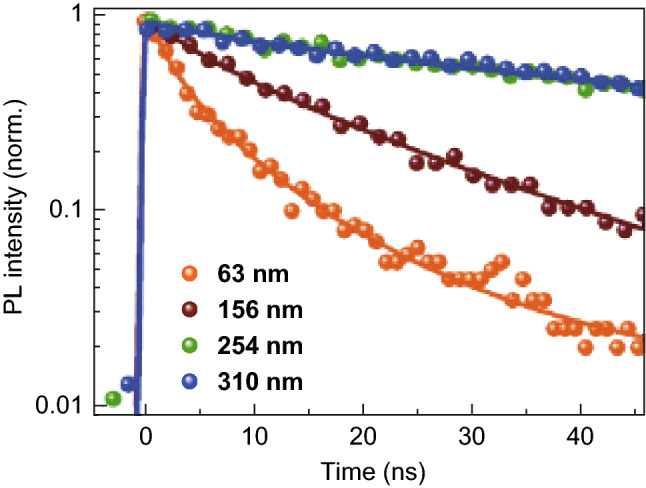
Thickness-dependent time-resolved PL data illustrate PL decay curve of CH_3_NH_3_PbI_3_ films dipped at different concentrations of PbI_2_ upon excitation at 517 nm—the solid line corresponds to the results that are stretched exponentially. Reproduced with permission Ref. [203]

**Fig. 21:**
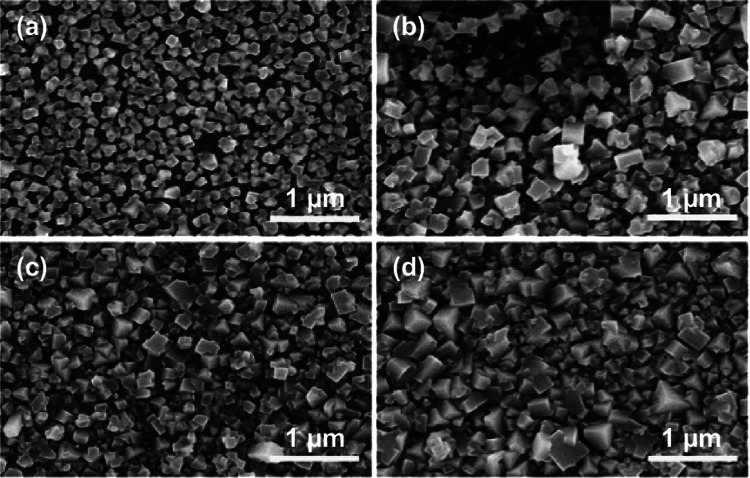
SEM images of CH_3_NH_3_PbI_3_ films of different PbI_2_ concentrations: **a** 0.3 M, **b** 0.5 M, **c** 0.8 M, and **d** 1.1 M. Reproduced with permission Ref. [203]

All these reports puzzled readers, but Yu Li attempted to clarify all the conflicts and observed direct charge transfer in perovskite [197, 203–205]. The thickness-dependent fluorescence lifetime could be found from the report of Li et al. published in *Nature* [203]. The results reveal that hole diffusion is faster than electron diffusion in the films, and it is dependent on the thickness of PbI_2_ by means of time-resolved transient fluorescence. They prepared all samples on glass substrate. A two-step chronological deposition method was underused to fabricate CH_3_NH_3_PbI_3_ perovskite in a nitrogen atmosphere. PbI_2_ film was spin-coated at different concentrations of *N*,*N*-dimethylformamide (DMF) at room temperature to obtain different thin-film thicknesses. Soon the films, which were dipped into CH_3_NH_3_I solution in 2-propanol at 65 °C for 90 min., dried, the films were annealed at 100 °C for 40 min. The resultant films were spin-coated with PCBM or PMMA for 1 h. Figure 20 shows that the transient fluorescence decays for the peak emission wavelength at 517 nm. The SEM images show that larger crystalline structures (250 nm) were observed in the case of thick films (Fig. 21d), and the grain growth is well in agreement with the Liu and Xiao reports. As the thickness increases, the relative amount of PbI_2_ reacting with the film decreases [23]. Smaller grains dramatically reduce the lifetime because of more crystal defects, but the larger thickness for the same increases the lifetime. Meanwhile, the defects at smaller crystals could be reduced by an adequate amount of PbI_2_ at the grain boundaries. Both factors are crucial in reducing the defects and increasing the fluorescence properties [8, 24, 206]. If mesoporous TiO_2_ and Al_2_O_3_ are used as substrate, that could reduce the grain growth. Overall, the fluorescence properties could be achieved for a thicker film of 390 nm and obtained diffusion distance of 1.7 μm for electrons and 6.3 μm for holes. For thin films, it could be approximately 95 μm, which may reduce the perovskite cell performance. Wang reported that the distorted crystal leads to the polarization of positive and negative ions, which produce dipole movement [207]. The polarization not only depends on oppositely charged ions but also on the special arrangements of ions in between the interstitial atomic planes. Much research on the polarization mechanism of electron–hole pairs is still needed.

## Lead-Free Perovskite Preparation and Characterization

Pb-based perovskite solar cells with a PCE exceeding 20% have been achieved, but the toxicity issue of Pb still remains a major problem for industrialization. The fabrication of perovskite films by solid-state spin coating on mesoporous anatase TiO_2_ in all the cases achieved a slightly higher result. Recently, Singh reported that the other perovskite without Pb (toxic) and Sn (poor stability to ambient condition) is methylalkylammonium iododibismuthate crystals (CH_3_NH_3_)Bi_2_I_9_. Although it showed less PCE and less toxicity, its stability was physically powerful for ambient conditions [208]. By trial-and-error methods, researchers may find a better conversion efficiency in the future.

Zuo et al. [58] demonstrated a family of perovskite materials, (NH_4_)_3_Sb_2_I_*x*_Br_9−*x*_, in ethanol, which is an eco-friendly solvent. The light absorption was tuned by adjusting I and Br contents. An attempt was made to replace Pb^2+^ by Sn^2+^ as a light absorber on mesoporous TiO_2_ CH_3_NH_3_SnI_3_ films. Due to the instability of CH_3_NH_3_SnX_3_ (X = Cl, Br, I) in ambient atmosphere, materials were placed in a nitrogen-filled glove box by dissolving equimolar quantities of SnI_2_ and CH_3_NH_3_I_3_ in degassed *N*-dimethylformamide (DMF), and then, the solution was spin-coated onto the substrate (FTO) at 2000 rpm for 45 s. The stability of as-synthesized material at ambient condition requires no heat treatment for crystallization, due to the oxidation of Sn^2+^ to Sn^4+^, resulting in the formation of oxides of Sn and methylammonium iodide [3, 209–211]. *I*–*V* results revealed the simulated diffusion length against photoexcited carrier concentration for five different doped hole concentrations. If the doping level in the CH_3_NH_3_SnI_3_ perovskite were decreased to the order of 10^15^ cm^−3^, it could result in a promising approach to enhance the carrier lifetime [212]. The crucial issue is that the stabilization of Sn material within the crystal structure should be suppressed from oxidation, thus reducing the doping level, which enhances the long-term performance. Both Pb-based and tin-based perovskite on TiO_2_ and Al_2_O_3_ film had power conversion efficiencies (η) of 11.5% and 15.0%, respectively. However, the Sn-based compounds were fabricated under a nitrogen atmosphere in glove box. Anatase TiO_2_ films were prepared by spin coating a solution of colloidal particles (20 nm in size) on previously deposited TiO_2_ by atomic layer deposition, which involved spin coating in a nitrogen glove box to avoid hydrolysis and oxidation of the tin perovskite in contact with air. The triarylamine derivative 2,2,7,7-tetrakis-(*N*,*N*-di-*p*-methoxyphenylamine)-9,9 spirobifluorene (Spiro-OMeTAD) was then applied as an HTM on top of the mesoporous TiO_2_. Lithium bis(trifluoromethylsulfonyl)imide and 2,6-lutidine were added to important dopants to increase the hole mobility. The thickness of the mesoporous TiO_2_ film was in the range of 350–370 nm [2–4, 59, 213]. The HTM penetrates into the remaining pore volume of the perovskite/TiO_2_ layer and forms a 200-nm-thick capping layer on top of the composite structure. A thin layer of gold was thermally evaporated for the back-contact electrode of the device. Recent execution of CH_3_NH_3_PbX (X = I, Cl, Br) perovskite absorbers with organic hole conductors such as 2,2,7,7-tetrakis (*N*,*N*-di-*p*-methoxyphenylamine) 9,9-spirobifluorene (Spiro-OMeTAD) converts PCEs greater than 15%, and this has been recognized as the “next big thing in photovoltaics.”

Lead-free perovskite of methylammonium tin iodide as the light-absorbing material was fabricated by solution-processed solid-state photovoltaic devices [214–216], which feature a lower optical bandgap of 1.3 eV than the 1.55 eV achieved with CH_3_NH_3_PbI_3_. Further alloying of iodide with bromide generates an efficient PCE of 5.8% under simulated full sunlight of 100 mW cm^−2^. The Sn-based perovskite obtains a higher symmetry phase (α-phase), even at room temperature, than Pb [217]. The compound has low carrier concentration and high electron mobility (*μ*_e_) on the order of ~ 1 × 10^14^ cm^−3^ and ~ 2000 cm^2^ V^−1^ S^−1^, respectively. This can be comparable or even superior to most traditional semiconductors, including Si, CuInSe, and CdTe. More importantly, the incident photon-to-electron conversion efficiencies (IPCEs) of the CH_3_NH_3_SnI_3_-based device cover the entire visible spectrum over 60% from 600 to 850 nm [217–219]. Thus, the efficiency of perovskite solar cells is not only related to the potential difference between the bands, but it can also be correlated with the energy difference between HTM potential and the conduction band edge (Table 8). Among the investigated CH_3_NH_3_SnI_3−*x*_Br_*x*_ perovskites, the device with CH_3_NH_3_SnIBr_2_ showed the highest PCE of 5.73%, with a *J*_sc_ of 12.30 mA cm^−2^, a V of 0.82 V, and an FF of 0.57. The absorption edge blueshift implies the reduction of *J*_sc_ with increasing Br content. Consistent with the bandgap tuning, the onset of the IPCE spectra blue-shifted from 950 nm for the iodide perovskite to 600 nm for the pure bromide perovskite.

**Table 8:** Optical bandgap and refined lattice parameter of the CH_3_NH_3_SnI_3−*x*_Br_*x*_ (*x* = 0, 1, 2, 3) perovskite and corresponding solar cell performance pattern

Perovskites	*E*_g_ (eV)	Lattice parameter	*J*_sc_ (mA cm^−2^)	*V*_oc_ (V)	FF	PCE (%)
CH_3_NH_3_SnI_3_	1.3	*a *= 6.169, *c *= 6.173	16.3	0.68	0.48	5.23
CH_3_NH_3_SnI_2_Br	1.56	*a *= 6.041 *c *= 6.053	14.38	0.77	0.5	5.48
CH_3_NH_3_SnI Br_2_	1.75	*a *= 5.948, *c *= 5.953	12.3	0.82	0.57	5.73
CH_3_NH_3_SnBr_3_	2.15	*a *= 5.837, *c *= 5.853	8.26	0.88	0.59	4.27

**Fig. 22:**
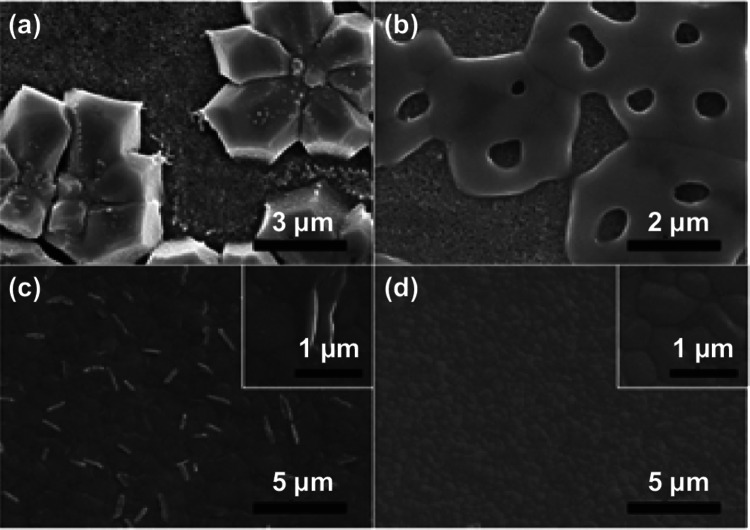
SEM images of FASnI_3_ perovskite film fabricated using *N*,*N*-dimethylformamide (DMF) solvent **a** without nonsolvent dripping and **b** with nonsolvent dripping; SEM images of FASnI_3_ perovskite film fabricated using mixed solvent of DMF and dimethyl sulfoxide (DMSO) with nonsolvent dripping, **c** the absence of pyrazine and **d** the presence of pyrazine. Reproduced with permission Ref.  [32]. Copyright ©2016 American Chemical Society

**Fig. 23:**
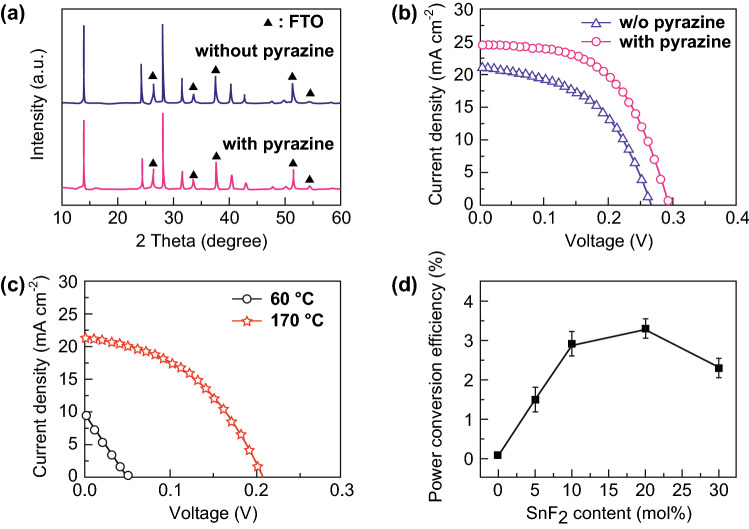
XRD patterns of FASnI_3_ films **a** with and without pyrazine deposited on FTO glass, **b** with and without the presence of SnF_2_ (10 mol%), **c** in the absence of SnF_2_ annealed at 60 and 170 °C, and **d** PCE of the FASnI_3_ and the amount of SnF_2_ added in the absence of pyrazine. Reproduced with permission Ref.  [32]. Copyright ©2016 American Chemical Society

**Fig. 24:**
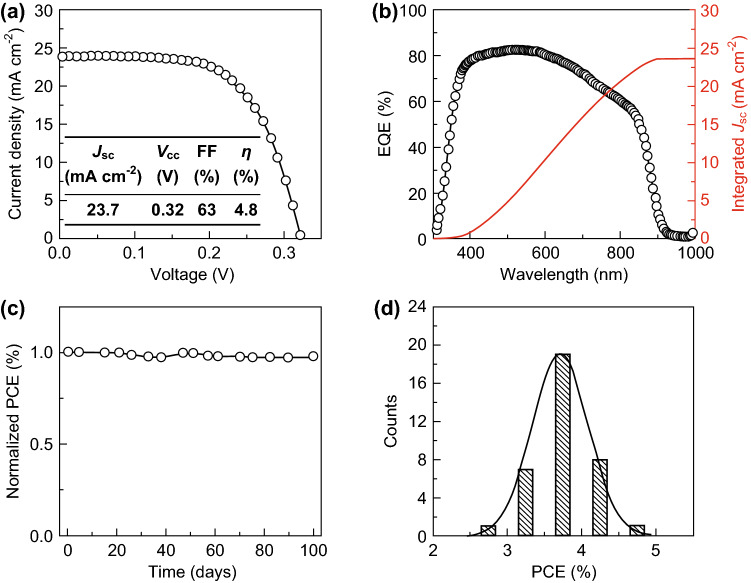
**a** *J*–*V* curve and **b** external quantum efficiency (EQE) spectrum and integrated *J*_sc_ for the optimized FASnI_3_ PSC; **c** recombination time constant of FASnI_3_ PSC_s_ as a function of the open-circuit voltage; and **d** relationship between PCE of the FASnI_3_ PSC and the amount of added SnF_2_ in the presence of pyrazine. Reproduced with permission Ref.  [32]. Copyright ©2016 American Chemical Society

Kanatzidis et al. synthesized a tin-based absorber on TiO_2_ films, and they also reported severe degradation against relative humidity under nitrogen atmosphere with poor reproducibility [199, 220, 221]. The results revealed a decrease in the conductivity due to the addition of SnF_2_, which reduces the Sn from Sn^2+^ to Sn^4+^. Recently, Koh et al. [5] reported the use of formamidinium tin iodide (FASnI_3_) as a light absorber with a bandgap of 1.41 eV at a broader temperature range up to 200 °C. A higher amount of SnF_2_ induces severe phase separation in the film aggregation in the improvement of the device [32, 222]. Aggregation can be reduced by the deposition of FASnI_3_ thin layer. Meanwhile, SnF_2_ also complicates this by accepting a lone pair from donors, such as triethylamine, 2,2-bipyridine, and 1,10-phenanthroline, including N atoms. A uniform FASnI_3_ layer is deposited using a nonsolvent dripping process by a one-step spinning process, revealing a very poor morphology (Fig. 22a). It can be improved by using a mixed solvent of DMF/DMSO (4:1) ratio. DMSO holds back the crystallization of FAI (Fig. 22b) and SnI_2_ and forms plate-like aggregates on the film resulting from excess SnF_2_ (Fig. 22c). Interestingly, the addition of pyrazine into solvent-engineered solution results in a uniform and dense FASnI_3_ perovskite layer (Fig. 22d). The layer annealed at 60 °C for 1 h provided a single phase of FASnI_3_ with a black color [32], and there is no significant peak from SnF_2_ in the film (Fig. 23a). Figure 23b presents the current–density–voltage (*J*–*V*) curves for those fabricated with and without pyrazine. In contrast, the PCE of a device fabricated in the presence of pyrazine in the mixture solution was improved to 4.0% (*J *= 24.5 mA cm^−2^, V = 0.29 V, and FF = 55%) as shown in Fig. 23b. In addition, the SnF_2_ is an important additive, which showed high efficiency at a higher temperature (Fig. 23c) [32]. At a higher temperature, Sn^4+^ will be eliminated, because Sn^4+^-related compounds can be evaporated at higher temperature [217, 223–226]. The PCE of FASnI_3_ PSC significantly increased as the SnF_2_ concentration increased from 3% with an addition of 10 mol% and then slightly improved to 3.3% at 20% of SnF_2_ (Fig. 23d). The efficiency of the device is also improved by controlling the thickness of each layer (Fig. 24a). The eternal quantum efficiency (EQE) spectrum for the best-performing solar cells is shown in Fig. 24b. Figure 24c shows that devices with pyrazine exhibit a longer recombination lifetime (*τ*_*n*_) than a reference device without pyrazine for all *V*_oc_ regions. The SnF_2_ incorporated into the Sn-based perovskite film is effective in suppressing the oxidation of Sn^2+^ to some extent. It is noticed that 10% SnF_2_ can yield the highest PCR in the pyrazine-treated FASnI_3_ PSC (Fig. 24d). The SnF_2_ was uniformly dispersed in the perovskite film by forming a complex with pyrazine with 4.8% efficiency [32, 197, 222, 227].

The toxicity issue has been tackled by the development of mixed-metal perovskite materials into bare tin analogues such as CH_3_NH_3_Sn_*x*_Pb_1−*x*_I_3_). Substituting the CH_3_NH_3_^+^ ion with HC(NH_2_)_2_^+^ gives CH(NH_2_)_2_PbI_3_ material that has a bandgap of 1.48 eV [227]. Most interesting, this material does not undergo any kind of phase transition, even though the temperature increased to 150 °C. It was synthesized by reacting stoichiometric amounts of formamidinium iodide and SnI_2_. The Tauc plot of formamidinium tin iodide shows an energy bandgap of 1.41 eV. The bandgap of formamidinium tin iodide is less than those of conventional perovskite CH_3_NH_3_PbI_3_ and formamidinium lead iodide (FAPbI_3_), which are 1.5 and 1.47 eV, respectively [203]. The stability of FASnI_3_ was also increased by the addition of 10, 20, 30, and 40 mol% of SnF_2_. The presence of fluoride was confirmed by XPS, and the data also reveal the presence of pure Sn^2+^ and not Sn^4+^. The surface morphology was improved by adding 10 mol% of SnF_2_ (Fig. 20a, b). As the concentration of SnF_2_ increased to 30 mol%, it was found to be a plate-like structure, which reduced the performance. However, the addition of 10 and 20 mol% of SnF_2_ into FASnI_3_ drastically increased the photocurrent density from 2.14 to 9.25 and 12.4 mA cm^−2^, respectively [203, 204]. The maximum PCE was achieved using 500-nm TiO_2_ thicknesses with a *J*_sc_ of 24.45 mA cm^−2^, a *V*_oc_ of 238 mV, and a fill factor of 0.36, yielding an overall PCE of 2.10%.

Recently, Tom et al. reported Pb-free cesium tin halide perovskites for utilization of the near-IR region [197, 204]. The results revealed that the tin-based compounds were red-shifted compared with Pb-based nanocrystals. Because of higher negativity, the Sn ion may occupy the B site in the ABX_3_ crystals. With the change in compositional content of the halide in the material, the absorbance band could be tuned. By adjusting the reaction temperature in the CsSnBr_3_ nanocrystals between 125 and 170 °C, the optical bandgap was also tuned from 630 to 680 nm. This is dependent on weak quantum confinement for a different shape and size of the CsSnBr_3_ perovskites. It was found that particles are more stable when prepared at low temperature. To obtain stable CsSnX_3_ perovskites, the tin precursors were dissolved in SnX_2_ in the mild reducing as well as coordinating solvents like tri-*n*-octylphosphine.

Cortecchia reported the synthesis of copper-based hybrid perovskite with the plan of investigating the film technology, optoelectronic properties, luminescence properties, and stability against copper reduction and enhance the perovskite crystallinity by changing the Br/Cl ratio. In reality, the Cu^+^ trap states could be responsible for an efficient green emission of these perovskites. The effects of annealing conditions were studied to optimize and to obtain crystalline and single-phase films. The optimal annealing condition for better crystallinity was found to be at a lower temperature (70 °C) for 1 h. As the temperature increases, the perovskite materials start degrading, and lower temperature and annealing time (70 °C for 30 min) also matter a lot for crystallization (Table 9). Incorporation of optically active cations takes a major role in overcoming the Pb-free perovskites [205]. A few years back, Zhao et al. [228] demonstrated an idea of cation transmutation to realistically design stable Pb-free halide perovskites for solar-cell applications. Transmuting two divalent Pb ions in APbX_3_^VII^ into one monovalent ion M^+^ (A_2_M^+^M^3+^V_6_^VII^), a double-perovskite structure can be formed. These materials usually show a broad absorption range and good phase stability against decomposition with assorted electronic properties. If carefully screening the periodic table, one could find approximately 11 nontoxic materials A_2_M^+^M^3+^X_6_^VII^ that are more promising for the Pb-free APbX_3_^VII^ perovskites. They show extremely high thermodynamic stability and suitable bandgaps. Theoretically, among them, CsInSbCl_6_ and Cs_2_InBiCl_6_ with *E*_g_ 1.0 eV show high PCE comparable to CH_3_NH_3_PbI_3_. There were good agreements on lattice parameters and bandgaps between theory and experiments. A few materials were obtained with A_3_Bi_2_I_9_ (A-MA or Cs) in investigations on crystal structure and optical, dielectric, and other chemical properties, but, in terms of efficiency, no practical applications have been done.

**Table 9:** Crystal structure and lattice parameters of Cu-based perovskites

Perovskite	Crystal system	*a*	*B*	*c*
MA_2_CuCl_4_	Monoclinic	7.2574	7.3504	9.9688
MA_2_CuCl_2_ Br_2_	Orthorhombic	7.3194	7.3281	19.1344
MA_2_CuCl Br_3_	Orthorhombic	7.3965	7.3686	19.3217
MA_2_CuCl_0.5_Br_3.5_	Orthorhombic	7.4276	7.4686	19.3075

Park et al. [214] reported low-temperature preparation of a one-step spin-coating method for MA_3_Bi_2_I_9_Cl_*x*_ followed by efficiency measurements. The crystalline phases were well assigned to the hexagonal phase, and the crystal growth preferred orientation was found to be at the *c*-axis (0 0 6), and this is rather different compared with bulk Cs_3_Bi_2_I_9_ of polycrystalline. The shifts in peaks were found to be at higher 2*θ*, which shows that a somewhat crystal structure has been affected by changing the cation, but still it is close to the hexagonal crystalline phase. As the crystalline structure changes, the optical band edge also changes to be approximately 2.1, 2.2, and 2.4 eV for the MA_3_Bi_2_I_9_, Cs_3_Bi_2_I_9_, and MA_3_Bi_2_I_9_Cl_*x*_, respectively. The fabrication process was done on FTO, and then, the TiO_2_ layer was deposited by spray pyrolysis on a hot plate. The OIHP precursor was also deposited by spin coating at 100 °C for 30 min. The OHT Spiro-OMeTAD was dissolved in chlorobenzene, and LiTFSI and tert-butylpyridine (TBP) were used as additives. BiI_3_ has caught the attention of many researchers recently for PV applications because of its tunable bandgap of 1.8 eV. Furthermore, BiI_3_ has an absorption coefficient > 10^5^ cm^−1^ in the longer-wavelength region. However, some of its properties may be resolved through further materials development and could be used for thin films in the future.

Based on these properties, Brandt et al. [215] reported materials preparation conducted by physical vapor deposition and solution processing. The BiI_3_ single crystals were grown using a modified vertical Bridgman method. To determine the potential of BiI_3_ as a photovoltaic absorber, it was crucial to grow a phase pure material [216]. Since the exact crystal structure of MBI was not yet reported, Eckhardt et al. [229] studied new bismuth-based materials, attempting to discover a new methylammonium iodobismuthate (CH_3_NH_3_)_3_Bi_2_I_9_. Methyl ammonium iodide and bismuth (III) iodide in DMF (3:1) were used to obtain pure phase with better crystal structure. The cell performance was low due to uneven morphology of the MBI layer and mismatch in structure. The results show that it is essential to gain more insight into carrier transport at the interfaces within the crystal structure. Sun et al. came up with a new and low‐cost hole selective material, 2,6,14‐tris(5′‐(*N*,*N*‐bis(4‐methoxyphenyl)aminophenol‐4‐yl)‐3,4‐ethylenedioxythiophen‐2‐yl)‐triptycene, i.e., TET, for efficient perovskite solar cells (PVSCs). As a 2,2′,7,7′‐tetrakis (*N*,*N*′‐di‐*p*‐methoxyphenylamine)‐9,9′‐spirobifluorene (Spiro‐OMeTAD) derivative, TET inherits excellent optoelectronic properties and overcomes the major shortfalls of Spiro‐OMeTAD [230, 231]. Li et al. worked on Pb-free pseudo-three-dimensional perovskites. Most of the complex iodobismuthate structures [Bi_2_I_9_]^3−^ are built from zero dimensions. Different cations were studied, such as CH_3_NH_3_^+^, NH_4_^+^, Cs^+^, Rb^+^, and K^+^ [232], which may be utilized for solar cell applications.

Organic units play an energetic role in intermolecular interactions and the frontier orbitals, which make them pseudo-three-dimensional networks of charge transfer units. In addition, they have been mentioned regarding cost-effective solar cells made up of carbon electrodes, achieving PCE of 0.9%. Red and block-shaped single crystals were grown using C_5_H_6_NBiI_4_ and C_6_H_8_NBiI_4_ dissolved in ethanol and a water system, respectively. The Bi–I bond lengths were shorter than bridging iodine atoms in each BiI_6_ and the bond angle for I–Bi–I ranged from 84.75° to 95.38°, which implies the angles of I–Bi–I in each bridging BiI_6_ octahedron are generally smaller than those of I (terminal) Bi–I, and this indicates the distortion of BiI_6_ in iodobismuthates. The stability performance was examined by XRD to prove there is no major change in the crystal structure, even after 1 week under ambient temperature in the dark. The slight change in the crystalline structure is due to the complete evaporation of solvent from the perovskite material. Without using HTM, the cell performance achieved a PCE of 0.9%; this explores the competition among bismuth-based solar cells [233, 234].

Saliba et al. [235] reported that most of the perovskite complexity is stimulated by the need to improve stability by incorporating more inorganic materials and increasing the entropy of mixing, which obviously makes unstable materials. A wider range of cations needed to be explored, with an appropriate Goldschmidt tolerance factor of between 0.8 and 1.0, which rendered the cations too small for consideration. The calculated tolerance factor data showed poor performance for CsPbI_3_ compared with other alkalis metals (Li, Na, K, Rb, Cs) and MA, FA, which are oxidation-stable monovalent cations. The ionic radium of Cs (167 pm) is marginally bigger than the Rb 152 pm. The small change in the crystal will have a large impact on optical properties, crystallinity, and stability. Both CsPbI_3_ and RbPbI_3_ were yellow at room temperature; upon heating to 380 °C, only CsPbI_3_ turned black, whereas RbPbI_3_ remained yellow [216]. As they increased to another level around 460 °C, both complexes started melting, and RbPbI_2_ turned black. Thus, Rb also has a black phase and desirable oxidation stability, but, so far, no one has used it for perovskite solar cells.

 The cell configuration of FTO/compact TiO_2_/mesoporous materials. TiO_2_/perovskite/Spiro-OMeTAD/Au. The charge transport within the RbCsMAFA (MA-methylammonium and FA-formamidinium) perovskite layer was substantially faster than in CsMAFA, and the device retained its 95% of stability under 85% relative humidity for 50 h with continuous irradiation of maximum solar intensity in a nitrogen atmosphere. The device started with > 17% efficiency at room temperature under a relative humidity condition in natural atmosphere [229]. The same kind of work was continued by Harikesh et al. [234] by selecting the elements that have the same electronic configuration as Pb^2+^ with outer *s* electrons as its replacement. The immediate possibilities are Bi and Sb that are nearer to Pb in the periodic table, which form +3 ions as Pb^2+^ does, and this hypothesis strengthens the idea of replacing Pb as Rb cations with Sb. Even though the photoconversion efficiency is less to understand more features, deeper insights into the defect characterization of the materials and optimizing the fabrication methodologies may make them better in the future [234].

**Fig. 25:**
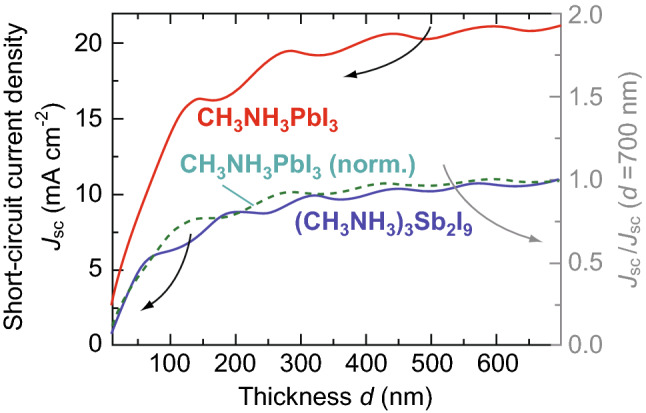
Plot of the thickness-dependent short-circuit current density *J*_sc_ of different perovskites. Reproduced with permission Ref.  [236]. Copyright ©2016 American Chemical Society

Hebig et al. [236] demonstrated the exchange of bismuth by antimony, which is less toxic than Sn and Pb, but the results on solar cell performance were so far rather poor. The (CH_3_NH_3_)_3_Sb_2_I_9_ thin films were fabricated via spin coating of a precursor solution (SbI_3_ and CH_3_NH_3_I in a mixture of butyrolactone and dimethyl sulfoxide followed by low-temperature annealing via spin coating). The XRD confirms strong preferential growth of (CH_3_NH_3_)_3_Sb_2_I_9_ along the c-axis. In addition, the Sb shows lower sub-bandgap absorption compared with Cs_3_Bi_2_I_9_, MA_3_Bi_2_I_9_, and FABi_2_I_9_, implying lower-density absorption states in the material than for Bi compounds. Layer absorbance was represented as ITO/PEDOT:PSS (25 nm)/perovskites/PC_16_BM (60 nm)/ZnO (60 nm)/Al. Figure 25 shows the thickness-dependent short-circuit current density. It is understandable by comparison between the Sb-based perovskites and Pb-based ones that there is a gradual decrease in the achievable photocurrent under solar illumination from lower to higher bandgap. At normalized short-circuit current density at 700-nm thickness, it becomes clear that Sb-based materials reach the absorption band as fast as Pb does, due to the absorption coefficient reaching high values close in energy to the absorption inception. The heterojunction device photoconversion efficiency was found to be 0.5%, and this could be much higher if the morphology, crystallinity, and contact layers were well aligned (Table 10). In addition, the atomic radii of Ge^2+^ are smaller than those of Pb^2+^, but it has similar outer ns^2^ electrons with low ionization energy, which may be useful to realize better optical absorption and carrier diffusion in the perovskite crystal. Ge-based perovskites are thermally stable up to 150 °C. The bandgap of CsGeI_3_ is 1.63 eV, which is very close to the conventional Pb-based perovskites [237]. The MAGeI_3_ and CsGeI_3_ show photovoltaic performance of 0.11% and 0.2%, respectively.

**Table 10:** Summary of recently reported improved lead-free perovskites

Cell configuration	Preparation method	*J*_sc_ (mA cm^−2^)	*V*_oc_ (V)	PCE (%)	Refs.
FTO/TiO_2_/CH_3_NH_3_SnI_3_/Spiro-OMeTAD/Au	Solution-based spin coating	16.8	0.88	6.4	[32]
FTO/TiO_2_/FASnI_3_/Spiro-OMeTAD/Au	Solution-based spin coating	24.45	0.238	2.1	[213]
FTO/TiO_**2**_/FASnI_3_/Spiro-OMeTAD/Au	One-step spinning process	23.7	0.323	4.8	[32]
FTO/TiO_2_/MASnI_3_/Spiro-OMeTAD/Au	Gas–solid reaction Vapor deposition	17.36	0.27	1.86	[216]
FTO/TiO_2_/Rb_3_Sb_2_I_9_/PolyTPD/Au	Spin coating	2.11	0.55	0.6	[229]
ITO/PEDOT:PSS/perovskites/PC_16_BM/ZnO/Al	Solution-processed spin coating	1	0.88	0.5	[230]

## Summary and Outlook

**Fig. 26:**
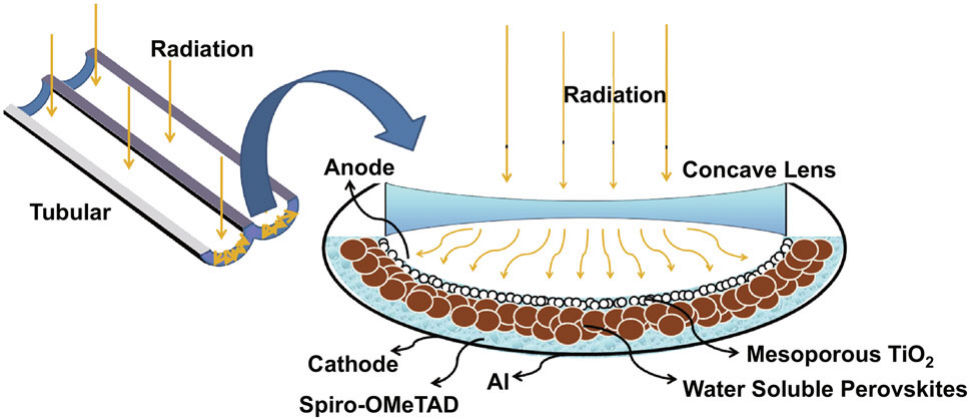
Cross-sectional image of as-proposed low-cost model for water-soluble perovskite solar cells

In this review, an overview of the perovskite materials’ growth with time to time has been compiled. For industrialization of large area modules, low-temperature vacuum deposition and CVD process were found to have similar efficiency of 16.5% because of better crystallinity, electron–hole transfer, optical absorption length, and charge-carrier diffusion length, and different vacuum techniques will definitely continue to be developed. As a decisive aspect, one-step vacuum deposition is superior in flexibility and for scaling up in practical applications. This technique gives insight into efficiently managing the environmentally hazardous Pb during processing, which significantly reduces the amount compared with all conventional and solution-processing methods. In addition to that, these materials degrade quite rapidly, even when one scans them taking an *I*–*V* curve. That will probably be one of the major challenges. A few studies on stability have been reported, and they identified the weak components in the phase structure. Some low-cost PV industry-friendly encapsulation schemes for perovskite solar cells have been tried. A simple encapsulation of PSC devices using quality flexible barriers can significantly extend their lifetime under ambient storage conditions. However, the use of halides in the perovskites provides abundant and inexpensive starting materials. In this connection, future research should aim at finding green synthesis of Pb-free, highly efficient, and environmentally friendly synthetic methods for perovskite materials. The higher stability of the organic cations analog could arise due to a more rigid perovskite structure from the enhanced hydrogen bonding between cations and an inorganic matrix. In addition, the atomic radii of Ge^2+^ are smaller than those of Pb^2+^, but they have similar outer ns^2^ electrons with low ionization energy, which is perhaps useful to get better optical absorption and carrier diffusion in the perovskite crystal. Ge-based perovskites are thermally stable up to 150 °C. The bandgap of CsGeI_3_ is 1.63 eV, which is very close to the conventional Pb-based perovskites. The small change in the crystal will have a large impact on optical properties, crystallinity, and stability. The results show it is essential to gain more insight into carrier transport at the interfaces within the crystal structure. However, the requirement of developing sustainable technologies based on nontoxic metals, particularly Fe^2+^, Cu^2+^, Zn^2+^, Sn^4+^, Ti^4+^, Sb^3+^, and Rb^+^, could be an alternative to Pb-based perovskites in terms of potential applications. In the periodic table, 70% of elements are most naturally abundant and could be obtained from the Earth’s crust, which are the most significant for life and, particularly transition, elements. There are approximately 52 cations and 13 anions, which are more favorable for the industrialization of materials. Theoretically, through permutation and combination for both the ions, it is possible to have almost one trillion (6.35013 × 10^11^) compounds to work out for a suitable technique in the field of solar cells. As for stability, perovskites react with H_2_O and start degrading (hydrated intermediate) followed by HI and PbI_2_. Recently, concentrated photovoltaic cells (CPVs) are able to convert 40% of incoming light into electricity by absorbing different wavelengths at the different heterojunction layers. However, the cost of CPVs is higher than that of the equivalent PV systems, because of curved mirrors and the tracking system. The concentrated high intensity may degrade the compounds or burn sometimes, and the cooling of films (heat up because of high radiation) and the maintenance of curved lenses from the dust lead to low power output. Concerning these facts, an idea for a simple, low-cost model called “dispersed photovoltaic cells” (DPVs) is highlighted. In CPVs, light is concentrated on a particular small area of thin films, but here one can use a concave lens to disperse the radiation on a large area of thin films. Because of dispersion or spreading, all the radiation will be absorbed by perovskite thin films. It is just as with solar water heater tubes, but the half large tubes of anode and cathode can be fabricated easily by any deposition technique. Concerning the moisture stability, in the future one can develop water-soluble perovskites for better stability. Figure 26 shows the cross section of a large DPV tube and layer-by-layer material fabrication. Because of the instability of the perovskites toward H_2_O molecule, perhaps in the future using this model, water-soluble perovskites could be synthesized for high moisture stability.
